# Moxibustion for pain relief in patients with primary dysmenorrhea: A randomized controlled trial

**DOI:** 10.1371/journal.pone.0170952

**Published:** 2017-02-07

**Authors:** Mingxiao Yang, Xiangzhu Chen, Linna Bo, Lixing Lao, Jiao Chen, Siyi Yu, Zheng Yu, Hongzhi Tang, Ling Yi, Xi Wu, Jie Yang, Fanrong Liang

**Affiliations:** 1 School of Acupuncture and moxibustion, Chengdu University of Traditional Chinese Medicine, Chengdu, Sichuan, China; 2 Pixian Hospital of Traditional Chinese Medicine, Chengdu, Sichuan, China; 3 Rentong Clinics of Traditional Chinese Medicine, Chengdu, Sichuan, China; 4 School of Chinese Medicine, The University of Hong Kong, Hong Kong; 5 Medical Center and Hospital of Qionglai, Chengdu, Sichuan, China; VU medisch centrum, NETHERLANDS

## Abstract

**Background:**

Though moxibustion is frequently used to treat primary dysmenorrhea in China, relevant evidence supporting its effectiveness is still scanty.

**Methods:**

This study was a pragmatic randomized, conventional drug controlled, open-labeled clinical trial. After initial screen, 152 eligible participants were averagely randomized to receive two different treatment strategies: Moxibustion and conventional drugs. Participants and practitioners were not blinded in this study. The duration of each treatment was 3 months. The primary outcome was pain relief measured by the Visual Analogue Scale. The menstrual pain severity was recorded in a menstrual pain diary.

**Results:**

152 eligible patients were included but only 133 of them eventually completed the whole treatment course. The results showed that the menstrual pain intensity in experimental group and control group was reduced from 6.38±1.28 and 6.41±1.29, respectively, at baseline, to 2.54±1.41 and 2.47±1.29 after treatment. The pain reduction was not significantly different between these two groups (*P* = 0.76), however; the pain intensity was significantly reduced relative to baseline for each group (P<0.01). Three months after treatment, the effectiveness of moxibustion sustained and started to be superior to the drug’s effect (-0.87, 95%CI -1.32 to -0.42, *P*<0.01). Secondary outcome analyses showed that moxibustion was as effective as drugs in alleviating menstrual pain-related symptoms. The serum levels of pain mediators, such as PGF_2α_, OT, vWF, β-EP, PGE_2_, were significantly improved after treatment in both groups (*P*<0.05). No adverse events were reported in this trial.

**Conclusions:**

Both moxibustion and conventional drug showed desirable merits in managing menstrual pain, given their treatment effects and economic costs. This study as a pragmatic trial only demonstrates the effectiveness, not the efficacy, of moxibustion for menstrual pain. It can’t rule out the effect of psychological factors during treatment process, because no blind procedure or sham control was used due to availability. In clinical practice, moxibustion should be used at the discretion of patients and their physicians.

**Trial registration:**

ClinialTrials.gov NCT01972906

## Introduction

Primary dysmenorrhea (PD) is prevalent among adolescent girls and women of reproductive age[1]. Its symptoms vary but typically include dull, throbbing and cramping pain in the lower abdomen during menstruation[2, 3]. Patients may also experience vomiting, nausea, diarrhea, fatigue, fever, headache, sleeplessness, and backaches[4]. PD usually starts from the onset of ovulatory cycles without any obvious underlying disease. While in secondary dysmenorrhea, there are usually substantial pathologies in the pelvic structure[5]. Primary dysmenorrhea typically begins before and is relieved soon after the onset of menstruation. The incidence of primary dysmenorrhea ranges from 45% to 72% of all menstruating women; however, among adolescent girls it can be as high as 93%[6]. In Modern medicine, it is believed that the excessive production and release of endometrial prostaglandin (PG) during menstruation may significantly induce uterine hypercontractility, reduce uterine blood flow, and trigger hypersensitive pain fibers[7]. Other studies suggest that menstrual cramp gets worse as PGF_2a_ increases and PGE_2_ decreases because the former pain mediator causes contraction and the latter relaxes the uterine smooth muscle[8]. So, an increase in the ratio of PGF_2a_ to PGE_2_ can be used as predictor of uterine contraction and thereby dysmenorrhea[9]. Moreover, the impact of PD on female health and life quality can be very considerable. Women with PD always suffer from severe physiological and psychological symptoms which greatly influence their quality of life and routine study[10, 11]. Studies found that PD is the leading cause of recurrent short-term school absenteeism among adolescent girls and a prevalent problem in menstruating women[12].

Drugs become very essential to relieve menstrual pain because of the huge impact of pain on patients’ life quality. Nowadays, treatment for primary dysmenorrhea includes a variety of pharmacological and non-pharmacological methods. Conventional pharmacological interventions include non-steroidal anti-inflammatory drugs (NSAIDs) and oral contraceptives[13]. According to a systematic review, the NSAIDs are effective to alleviate PD symptoms [3]. Pharmacological treatment can provide pain relief, but one common argument is that NSAIDs are frequently associated with a variety of adverse effects that includes gastrointestinal disorders, nephrotoxic and hepatotoxic effects, and fluid retention[13]. Furthermore, women with PD might have (beginning) endometriosis. NSAIDs can provide pain relief but no prevention of disease progression. Postoperative use of continuous oral contraceptives has been shown by a recent systematic review to be associated with a reduction in the recurrence rate of dysmenorrhea, delay in the presentation of dysmenorrhea, reduction in nonspecific pelvic pain, and reduction in the recurrence rate for endometrioma[14]. However, it is noteworthy that patients who discontinued medication experienced a higher incidence of recurrence, indicating that the protective effect of these medications seems to vanish rapidly after the discontinuation[15]. Moreover, it has been reported that NSAIDs fail to alleviate menstrual pain in about 20% of women[16]. Therefore, the search of an alternative and effective non-pharmacological intervention to relieve menstrual pain represents urgent clinical demands. In China, pharmacological methods for PD are not limited to conventional drugs. Actually, more and more young PD patients frequently resorted to traditional Chinese medicine for pain relief[17].

In addition to herbal decoctions, traditional Chinese medicine therapies include a number of effective interventions such as acupuncture, moxibustion, Tuina/massage, auricular acupuncture, acupressure, etc. Moxibustion therapy as the combination of pharmacological material and non-pharmacological practices is commonly used in clinics or by patients themselves to treat primary dysmenorrhea. As a treatment strategy that is associated with physical touch and verbal interactions, this therapy undoubtedly has psychological effects on patient. In addition to that, the effectiveness of moxibustion mainly comes from patients’ physiological responses[18, 19] to heat stimulation generated by burning moxa, and chemical stimulation[20, 21] of the pharmaceutical components in mugwort leaves. Thus, possible underlying mechanisms of moxibustion can be explained by these temperature-related and non-temperature-related factors. According to a recent review, the heat stimulation of moxibustion can activate inflammatory responses and induce vascular change[22].

Though empirical studies and theoretical explanations show that moxibustion is effective for treating PD, the good quality evidence is still scanty according to recent systematic reviews[23–25]. A review included 20 RCTs with 2134 participants to assessed the effects of moxibustion or acupoint therapy for the treatment of PD. These studies all suggested that moxibustion induced menstrual pain recovery, however, the evidence quality was unsatisfactory due to small sample size and lack of randomization, etc. Therefore, we performed this pragmatic randomized, conventional drug controlled, clinical trial to assess the effectiveness of moxibustion for PD.

## Materials and methods

### Ethical approval and trial registration

**Fig 1:**
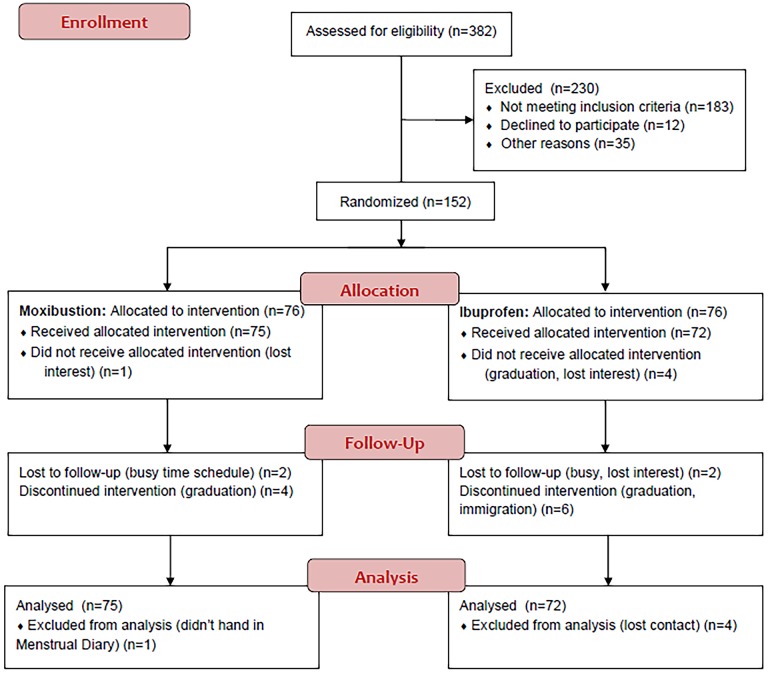
Study flow chart. This study is a randomized, drug-controlled, open-labeled clinical trial. 152 eligible patients were randomly assigned to either the moxibustion group or the drug-control group. After treatment for 3 months, menstrual pain intensity measured by VAS and menstrual intensity were assessed to evaluate the effectiveness of moxibustion for pain relief as compared with painkillers. Menstrual pain-associated serum markers/chemical compounds were tested as another secondary outcome. The follow-up lasted for 3 months after treatment.

This trial is a pragmatic randomized, open-label, drug-controlled clinical trial that compared two different treatment strategies (moxibustion versus conventional drug) (Fig 1). All trial procedures have been ethically reviewed and approved by Sichuan Regional Ethics Review Committee on Traditional Chinese Medicine (2013KL-004) (S1 File). All patients provided written informed consent before the trial started. This trial was registered after recruitment due to the change of research managers. The authors confirmed that all ongoing and related trials for this intervention were registered. Study protocol of this study was published online[6](S2 File).

### Participant recruitment

According to sample size calculation, 152 PD patients were recruited through campus advertisements from Chengdu University of Traditional Chinese Medicine, Southwest Jiaotong University, Southwest University of Nationalities, Southwestern University of Finance and Economics and several other universities in Chengdu. The implementation of this trial has no violation to its protocol. We originally intended to analyze all blood samples, however, only 30 blood samples from each group were eventually analyzed due to funding limit. We received the ethic approval from authorities on February 1^st^, 2013. Patient recruitment phase ranged from March 1^st^, 2013 to August 12^th^, 2013. Post-treatment follow-ups started in June 15^th^, 2013 and ended at February 18^th^, 2014.

This study employed the diagnostic standards of the Clinical Guideline of Primary Dysmenorrhea by the Society of Obstetricians and Gynecologists of Canada[26]. Patients matched the following inclusion criteria were considered eligible: (1) being aged from 18 to 35 years; (2) with a history of regular menstrual cycles (28 days±7 days); (3) having experienced menstrual pain of intensity from moderate to severe and the visual analog scale (VAS) ≥40 mm for at least 3 menstrual cycles before this study; (3) the syndrome differentiation of traditional Chinese medicine correlating Qi-stagnation and blood stasis syndrome and congealing cold-damp syndrome; and (4) providing a hard-copy of informed consent form. Moreover, patients met anyone of the following exclusion criteria were excluded: (1) women with secondary dysmenorrhea caused by endometriosis, pelvic inflammation, or myomas of uterus confirmed by type-B ultrasound exam by gynecologists; (2) women with irregular menstrual cycles; (3) women with uncontrolled neurological diseases, immunodeficiency, bleeding disorders, and allergies; (4) women with uncontrolled medical conditions which contraindicate moxibustion; (5) women taking prostaglandin synthetase inhibitor (PGSI) two weeks before inclusion; (6) women in lactation, pregnant women, or those with plans to get pregnant in the coming half year; (7) women taking drug such as NSAIDs or oral contraceptive pills that can influence the outcomes; (8) women receiving moxibustion currently or received moxibustion 2 weeks prior to enrollment; and (9) women undergoing other trials.

### Randomization and blinding

All physical examination and routine test were done in the 3^rd^ teaching hospital of Chengdu University of Traditional Chinese Medicine. After a primary assessment, baseline information and clinical characteristics of patients were collected in the 3-month baseline period. Then, ineligible participants were screened out. Baseline information and clinical characteristics of included participants were collected and categorized by a patient coordinator. Baseline data was then separated by a data processor and only demographic data was handed over to a 3^rd^ researcher, who was responsible to randomly allocate the patients to specific groups in a 1:1 ratio, according to a random digit table. The 3^rd^ researcher had no access to the recruitment, treatment, assessment process, or the other data. The numbered sealed opaque envelope was used to keep the randomization code, and was not disclosed to other researchers until the statistical analysis has been completed by statisticians. This study compared the effect of moxibustion therapy with conventional analgesics. Those two treatment strategies were so different that it’s hardly possible to blind the patients and practitioners. Thus, patients and clinical doctors were not blinded in this trial. However, data collectors and statisticians were blinded to the setting and treatments of different groups.

### Treatment strategies

Patients received different treatment strategies according to the group they were assigned to. For the conventional drug control group, patients were instructed to administrate Ibuprofen Sustained Release Capsules (Fenbid, 0.3g/capsule*12 capsules, Sino-GlaxoSmithKline, Tianjin, China). For the moxibustion group, two different TCM patterns of acupoints are selected for treatments (including diagnostic pattern 1: Qi-stagnation and blood stasis, and diagnostic pattern 2: congealing cold-camp). The acupoints for moxibustion treatment was based on data mining [27, 28] from literature and expert opinions. Guanyuan (CV4), Shenque (CV8), and Sanyinjiao (SP6) were selected as key acupoints receiving moxa heat stimulation. Mild moxibustion including moxibustion without cutaneous contact was used together with moxa roll which was made of dry mugwort leaves with a paper cylinder (Z32021062, Oriental Moxa Co., Suzhou, China). The participant was asked to perform the treatment in a comfortable supine position and the skin of every acupoint was sterilized. The ignited moxa roll was applied approximately 2–3 cm above the dermal layer of acupoints. Moxibustion treatment was conducted on the acupoints CV4 and CV6 at the same time. Right after that, the SP6 at both sides of the body were stimulated simultaneously. A mild warm and comfortable sensation that was quite similar to a ‘Deqi’ sensation in acupuncture was achieved before the skin was provoked with hyperemia. Moxibustion at each point commonly lasted for about 10 to 15 minutes. The entire treatment process for each patient lasted for about 25 to 30 minutes. Moxibustion treatment started 7 days before the beginning of menses and didn’t stop until the onset of next menstruation. Moxibustion practitioners (XC&SY) in this trial were licensed TCM doctors and had over 5-year experiences of the clinical practice. Participants in moxibustion group received moxibustion treatment once a day, 7 days a session for 3 sessions over 3 menstrual cycles.

**Fig 2:**
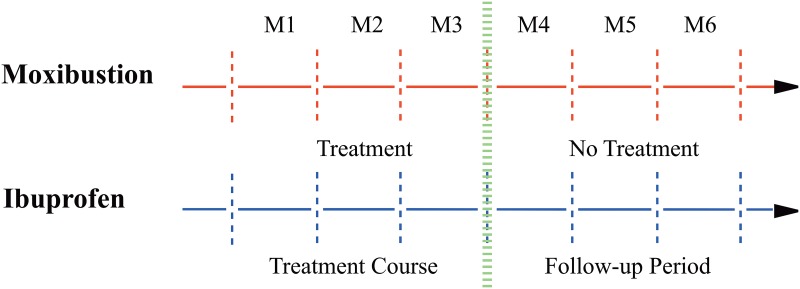
Timeline of treatment and follow-up. For both groups, the treatment course lasted for three months (M1-M3). After the completion of treatment, there’s a three-month follow-up period (M4-M6).

All the participants in the control group were instructed to use the Fenbid for pain relief. They took 0.3g capsules per time, twice a day for three menstrual cycles. Every session was one day before every menstrual cycle, lasting 3 days. Moreover, rescue painkillers were allowed for severe pain condition that exceeds patient’s endurance. The quantity and time of painkiller pills taken during the menstrual period were documented in the case-report form (CRF). The timeline of treatment and follow-up was shown in Fig 2.

### Outcome measurements

The effectiveness of moxibustion for pain relief on PD was assessed by the primary outcome: change from baseline in menstrual pain intensity measured by VAS at each menstrual cycle. Participants were asked to indicate a perception of pain intensity scored from 1 to 10 (0, no pain; 10, maximum) along a 100 mm horizontal line. The other outcome measures were the COX menstrual symptom scale at baseline and at the fourth menstrual cycle (after completion of three sessions of treatment) and lab test parameters (prostaglandin F_2α_ (PGF_2α_), oxytocin(OT), vWF, β-endorphin (β-EP) and PGE_2_). Safety of moxibustion was assessed by the occurrence of adverse events, such as burnt, allergy, scald, faint and others that have been recorded in the CRF.

### Sample size estimation

A power analysis (two-sample t-test, performed in G*Power software) showed that a sample size of 132 women would detect a difference in pain reduction of 2 cm weighed by the visual analogue scale (α = 0.05 and 1−β = 0.90). Thus, a total of 152 participants were anticipated in this trial for a compensation to 15% dropout rate, with 76 patients in each group.

### Statistical analysis plan

Data analysis was performed by statisticians who were blinded to the group settings. The SPSS software (SPSS 16 for Windows) was used to perform the data analysis. The analyses used both ‘intention-to-treat’(ITT) and ‘per-protocol’(PP) strategies [29]. Mean and standard deviations of the VAS scores, the COX dysmenorrheal symptom score were compared among participants who were treated with moxibustion or drugs. An independent-sample t-test was used to examine differences between moxibustion group and Ibuprofen group. Differences in the primary study outcome measures between the two groups were analyzed using linear mixed model analysis of variance with group and visit time as fixed effects, subject as a random effect for continuous, normally distributed variables and generalized estimating equations for categorical variables. Adjustments for the imbalance between the two groups were made for body mass index (BMI). *P*<0.05 was considered statistically significant (two-sided).

## Results

### Trial recruitment and subject flow

382 women were recruited for eligibility assessment. 230 of them were excluded due to not meeting inclusion criteria (n = 183), declining to participate (n = 12) and other reasons (n = 35). Therefore, 152 participants were included in this trial. 76 of them were allocated to moxibustion group. One later withdrew due to losing interest (n = 1). The remainder was in the control group. Four withdrew due to graduation (n = 3) and one due to losing interest (n = 1).

In the follow-up stage, two subjects in moxibustion group withdrew due to busy schedule and four discontinued their intervention as a result of graduation. For the control group, two lost to follow-ups due to busy work and reduced interest, six discontinued their intervention for the reason of graduation. At last, 133 participants (69 women in moxibustion group and 64 women in Ibuprofen group) completed all trial procedures and finally handed in all questionnaires. In analysis, 147 patients were included in ITT analysis due to the availability of baseline data (Fig 1).

### Background characteristics

All subjects were from Chengdu, the southwest part of China. Most of them were single (97%), non-smoker (100%), non-alcoholic (100%), and 90% were college students. The two groups were comparable in most baseline characteristics (Table 1). Baseline data showed well-balanced clinical features between groups except for BMI (Moxibustion group (Mean±SD) vs. Control group (Mean±SD):19.42±1.55 vs. 20.01±1.94, *P* = 0.043), therefore primary outcome was adjusted for BMI in the analyses.

**Table 1:** Baseline characteristics for participants.

Clinical characteristics	Moxibustion group, n = 75	Control group, n = 72
Age	23.01±2.98	23.09±2.88
BMI		
Underweight	28 (37.3%)	13 (18.1%)
Normal	47 (62.7%)	59 (81.9%)
Smoker	0	0
Alcoholic	0	0
Finished high school	0	2 (2.78%)
Currently undergraduate students	49 (65.33%)	45 (62.50%)
Currently graduate students	20 (26.67%)	21 (29.17)
Completed tertiary education	6 (8.00%)	4 (5.56%)
Pain intensity measured by VAS	6.38±1.28	6.41±1.29
TCM syndrome patterns		
congealing cold-camp	52 (69.33%)	47 (65.28%)
*Qi* stagnation and blood stasis	23 (30.67%)	25 (34.72%)
Menstrual pain duration (days)	5.38±2.79	5.63±2.88
Menstrual symptoms		
Intensity score measured by CMSS	16.01±7.96	15.78±6.90
Duration measured by CMSS (days)	20.64±11.07	19.83±9.22

### Primary outcomes

**Fig 3:**
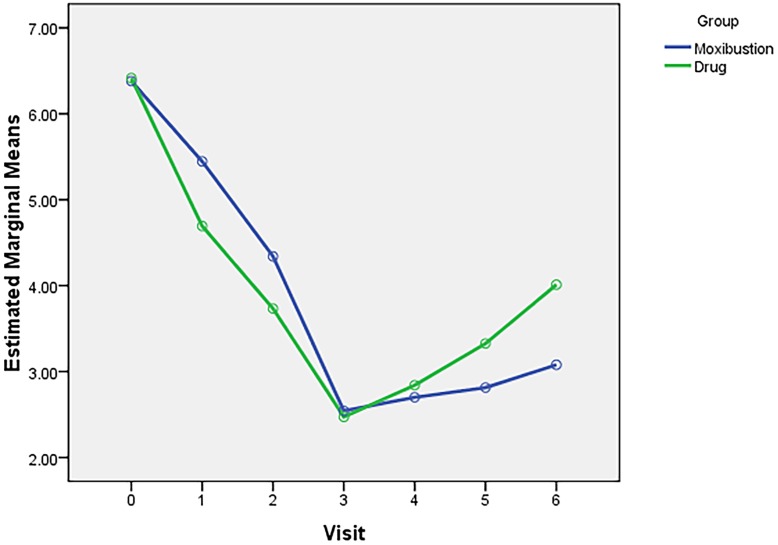
Pain intensity change at different time point. By employing a 3-month moxibustion treatment, menstrual pain intensity and its related symptoms were significantly improved. In the treatment course (the 1^st^ and 2^nd^ month), drug overweighed moxibustion treatment in terms of controlling pain severity, but at the end of the 3-month treatment, the improvement in moxibustion group was not significantly different to the drug control group. Moreover, the effect of moxibustion sustained to 3 months after treatment.

Results of adjusted and unadjusted ITT analyses were reported for primary outcome analysis (Table 2). During the treatment course (the 1^st^ month and the 2^nd^ month), the menstrual pain intensity measured by VAS was significantly reduced in the control group, as compared with moxibustion group(*P*<0.001). While, at the end of the 3^rd^-month treatment, menstrual pain intensity showed a significant reduction in both groups, there was no significant difference between moxibustion group and drug control group (0.07, 95%CI -0.38 to 0.52, *P* = 0.76). Moreover, the effect of moxibustion sustained to 3 months after treatment (Fig 3). At the 6^th^ month after randomization, the pain intensity in moxibustion group was significantly lower than that of the control group (-0.87, 95%CI -1.32 to -0.42, P<0.001). Results of PP analyses were included in the **Supporting Information (**S3 File**)**.

**Table 2:** Primary and secondary study outcomes by treatment group.

Menstrual outcomes	Moxibustion group, n = 75	Control group, n = 72	Unadjusted difference (95%CI)	Unadjusted *P*-value	Adjusted difference (95%CI)	Adjusted *P*-value
**Pain intensity measured by VAS**						
0 month	6.38±1.28	6.41±1.29	-0.04 (-0.45, 0.38)	.87	-0.04 (-0.46, 0.39)	.86
1 month	5.45±1.34	4.69±1.21	0.75 (0.34, 1.71)	<.0001	0.75 (0.33, 1.17)	<.0001
2 month	4.34±1.22	3.73±1.24	0.61 (0.21, 1.01)	<.0001	0.62 (0.21, 1.03)	<.0001
3 month	2.54±1.41	2.47±1.29	0.07 (-0.37, 0.51)	.75	0.07 (-0.38, 0.52)	.76
4 month	2.70±1.54	2.84±1.46	-0.14 (-0.63, 0.35)	.57	-0.12 (-0.61, 0.38)	.65
5 month	2.81±1.53	3.33±1.35	-0.51 (-0.98, -0.42)	.03	-0.46 (-0.94, 0.01)	.06
6 month	3.08±1.49	4.01±1.21	-0.93 (-1.38, -0.49)	<.0001	-0.87 (-1.32, -0.42)	<.0001
**Menstrual pain duration (days)**						
0 month	5.38±2.79	5.63±2.88	-0.24 (-1.16, 0.69)	.61	-0.34 (-1.28, 0.60)	.47
1 month	1.81±1.02	1.83±1.51	-0.03 (-0.45, 0.39)	.89	-0.10 (-0.52, 0.32)	.65
2 month	1.52±0.98	1.61±1.67	-0.09 (-0.54, 0.35)	.68	-0.14 (-0.59, 0.31)	.53
3 month	1.29±1.06	1.43±1.70	-0.14 (-0.60, 0.32)	.55	-0.18 (-0.65, 0.29)	.44
4 month	1.05±0.92	1.18±1.52	-0.13 (-0.54, 0.28)	.52	-0.17 (-0.59, 0.24)	.41
5 month	1.11±0.89	1.33±1.48	-0.23 (-0.62, 0.17)	.27	-0.25 (-0.66, 0.15)	.22
6 month	1.23±0.89	1.63±1.47	-0.40 (-0.79, 0.00)	.05	-0.43 (-0.83, -0.03)	.04
**Menstrual symptom intensity score measured CMSS**						
0 month	16.01±7.96	15.78±6.90	-0.24 (-2.20, 2.67)	.85	0.26 (-2.22, 2.73)	.84
1 month	14.07±7.92	12.79±6.13	1.28 (-1.04, 3.59)	.28	1.28 (-1.07, 3.64)	.28
2 month	10.85±6.49	10.76±6.24	0.09 (-1.99, 2.17)	.93	0.03 (-2.08, 2.14)	.98
3 month	6.24±4.99	7.65±5.26	-1.41 (-3.08, 0.26)	.10	-1.53 (-3.23, 0.16)	.08
4 month	6.12±4.95	7.71±5.25	-1.59 (-3.25, -0.07)	.06	-1.66 (-3.35, -0.03)	.05
5 month	5.21±4.55	8.58±5.47	-3.37 (-5.01, -1.73)	<.0001	-3.45 (-5.12, -1.79)	<.0001
6 month	4.84±3.19	9.78±5.38	-4.94 (-6.51, -3.37)	< .0001	-4.98 (-6.58, -3.39)	<.0001
**Menstrual symptom duration score measured CMSS (days)**						
0 month	20.64±11.07	19.83±9.22	0.81 (-2.52, 4.14)	.63	0.58 (-2.80, 3.96)	.74
1 month	16.37±8.85	15.53±7.82	0.85 (-1.88, 3.57)	.54	0.69 (-2.08, 3.46)	.69
2 month	12.56±8.51	12.61±7.53	-0.05 (-2.67, 2.57)	.97	-0.20 (-2.87, 2.47)	.88
3 month	7.03±5.97	8.78±6.30	-1.75 (-3.75, 0.25)	.09	-1.98 (-4.00, 0.05)	.06
4 month	6.44±5.48	8.25±5.75	-1.81 (-3.64, 0.02)	.05	-1.95 (-3.81, -0.09)	.04
5 month	4.91±3.88	8.10±5.35	-3.19 (-4.71, -1.67)	<.0001	-3.28 (-4.82, -1.74)	<.0001
6 month	4.51±3.73	9.40±5.36	-4.89 (-6.40, -3.39)	<.0001	-4.99 (-6.52, -3.47)	<.0001

### Secondary outcomes

There was no significant difference in menstrual pain durations (days) between two groups in the first 6 months (*P*>0.05). Three months after treatment (the 6^th^ month), the days with menstrual pain in moxibustion group was significantly less than that of the control group (-0.43, 95%CI -0.83 to -0.03, *P* = 0.04). Moreover, the severity of menstrual symptoms measured by CMSS showed a significant reduction for each group from month 0 to month 6. But quite similar to the trends of pain intensity and menstrual pain durations, the improvement of menstrual symptoms in moxibustion group was greater than the control group at the 6^th^ month after randomization (-4.98, 95%CI -6.58 to -3.39, *P*<0.01). Both treatments significantly reduced the duration of menstrual symptoms in the treatment course as well as the follow-up period. But the improvement in moxibustion group was different to the control group since the 4^th^ month to the 6^th^ month after randomization (Table 2).

The laboratory outcomes of blood test demonstrated that compared with baseline, both treatments significantly regulated serum PGF_2α_, OT and vWF, increased serum β-endorphin level (*P*<0.05). Additionally, moxibustion treatment significantly decreased serum PGE_2_, but such reduction was not detected in the control group. As to others, there was no significant difference observed between those two groups (Table 3).

**Table 3:** Changes of PD-related markers in serum.

Blood test	Experimental group, n = 30	Control group, n = 30	Unadjusted effect (95%CI)	Unadjusted *P*-value	Adjusted effect (95%CI)	Adjusted *P*-value
**Baseline**						
PGF_2α_ ↓	731.55±149.37	732.42±154.22	-0.86 (-79.33, 77.60)	.98	-3.13, (-84.40, 78.14)	.94
OT ↓	155.38±28.21	165.32±29.58	-9.94 (-24.88, 5.00)	.19	-11.06 (-26.48, 4.37)	.16
vWF ↓	2.72±0.89	2.69±0.74	0.04 (-0.39, 0.46)	.87	0.00 (-0.43, 0.43)	.99
β-EP ↑	206.27±68.12	226.60±81.65	-20.33 (-59.19, 18.53)	.30	-19.71 (-59.98, 20.56)	.33
PGE_2_ ↓	591.12±243.16	549.04±260.43	42.08 (-88.13, 172.30)	.52	39.22 (-95.68, 174.13)	.56
**3 months**						
PGF_2α_ ↓	311.88±110.53	298.74±81.86	13.14 (-37.13, 63.40)	.60	13.63 (-38.46, 65.72)	.60
OT ↓	65.32±25.34	61.01±20.29	4.31 (-7.56, 16.17)	.47	4.36 (-7.93, 16.66)	.48
vWF ↓	1.13±0.34	1.01±0.37	0.13 (-0.06, 0.31)	.17	0.13 (-0.06, 0.32)	.18
β-EP ↑	412.17±70.74	431.39±125.31	-19.22 (-71.80, 32.37)	.47	-17.19 (-71.64, 37.26)	.53
PGE_2_ ↓	332.11±211.39	454.26±213.45	-122.14(-231.93, -12.35)	.03	-99.57 (-210.28, 11.13)	.08

Additionally, during the treatment courses, four participants in moxibustion group and two patients in the control group took additional painkillers in the 1^st^ month after randomization. In the 2^nd^ months, the number of participants who took additional analgesics was reduced to three in the moxibustion group and two in the control group. In the 3^rd^ month, there were no patients took additional drugs to control menstrual pain. No adverse events were reported from both groups.

## Discussion

In this randomized controlled trial, we included 152 participants and assessed the effect of moxibustion therapy for PD by comparing with conventional treatment strategy. The results showed that by employing a 3-month moxibustion treatment, menstrual pain intensity and its related symptoms can be significantly improved. During the 3-month treatment course, drug overweighed moxibustion treatment in terms of controlling pain severity, but at the end of the treatment course, the improvement in moxibustion group was not less than that of the drug control group. Moreover, the effect of moxibustion sustained to 3 months after treatment completion. Other outcomes such as severity and duration of menstrual symptoms showed similar trajectory of improvements for two groups in different time courses. Supplementary blood test demonstrated that both moxibustion and drugs significantly regulated serum pain mediators.

For patients with PD, pain is the most critical symptom and the key reason to their reduced life quality and imbalanced emotional wellness. According to a recent epidemiology review, PD can cause severe pain, which is defined in different studies as pain requiring bed rest and missing work or limiting daily activity, or 8–10 points in a 10-points visual analogue scale (VAS)[30]. Another pilot study showed that PD is as intense as renal colic pain and thus deserves the same level of care and attention in emergency room as patients with renal colic pain get[31]. In recent decades, increased attention has recently been paid to non-pharmacological treatment for dysmenorrhea[32, 33]. In China, moxibustion among many other treatment options is frequently employed by clinical doctors or by patients themselves to manage menstrual pain. However, current evidence of moxibustion treatment for PD is still scanty. A current meta-analysis analyzed 20 randomized controlled trials (RCTs) with 2134 participants and found only moderate level evidence to support the benefits of acupuncture or moxibustion for PD[23, 34]. Another systematic review[35] by Cho SH et al. included 27 clinical trials of acupuncture for primary dysmenorrhea. Their study indicated that acupuncture is a promising tool for managing menstrual pain, but the quality of current evidence is limited by the methodological flaws. However, it should be pointed out that according to its searching strategy this review only focused on the effect of acupuncture, which is a sister treatment of moxibustion. It is true that acupuncture and moxibustion shared similar treatment principles such as acupoint-meridian theory, Qi-blood (energy) theory, but the nature of their stimulus is quite different from one another. In general, acupuncture in forms of manual or electrical techniques generates mechanical or electrical impulses, but moxibustion, on the other hand, mainly yields heat stimuli. Quite importantly, the study results of these two forms of alternative techniques are not interchangeable[36]. Therefore, we believe that it is inappropriate for current systematic reviews to combine acupuncture and moxibustion together for the purpose of enlarging sample size, to testify the effect of either of them. On the other hand, we argue that the result of one treatment (like acupuncture) shouldn’t and yet cannot be generalized to the other treatment (moxibustion), because they are clearly different in nature. Hence, to our knowledge, at present there is no sophisticated body of evidence to support or refute the effectiveness of moxibustion for menstrual pain. And one of the most significant reasons is the lack of pieces of evidence or relevant clinical trials—not even to mention trials with good methodology. Therefore, our study was conceived to help bridge this gap.

In the current study, we employed a pragmatic design to assess the effectiveness of moxibustion treatment for PD by comparing two treatment strategies. We found that moxibustion therapy significantly lowered menstrual pain intensity three months after treatment compared with baseline (Baseline VS the 3^rd^ month: 3.84, 95%CI 3.51 to 4.17, *P*<0.01), the therapeutic effect of moxibustion was not significantly different to the control group (Experimental VS Control: 0.07, 95%CI -0.37 to 0.51, *P* = 0.75). This suggests that moxibustion is not inferior to conventional drugs for treating PD. Due to the low availability of similar study, we only identified a few observational studies[37–42] that assessed the effect of moxibustion independently for PD and found that this result is in consistency with them. Lee’s study found that after moxibustion, the graphic rating score of menstrual cramps was decreased significantly from 7.79 (SD = 1.22) to 4.47 (SD = 2.25) in the experimental group[38]. Gao’s study suggested that 5–7 days after moxibustion treatment pain intensity was significantly lowered from baseline (5.37±2.0) to the 1^st^ menstrual cycle (3.2±1.7)[39]. For those two studies, the effect of moxibustion was significantly different to that of the placebo control. In this study, we applied a positive drug control instead of an inert one, for the reason that it is not our priority to assess the efficacy of moxibustion for PD. The pragmatic design enabled us to test the effectiveness of moxibustion as a treatment strategy for PD in a more real world environment[43–45]. By this design, we found that 3-month moxibustion treatment is neither superior nor inferior to drug treatment. Therefore, moxibustion therapy can be as effective as drugs for pain relief and should be recommended to patients with PD for pain relief.

Moreover, from the secondary outcomes we observed that in changing days with menstrual pain moxibustion and the drug didn’t differ from each other. Three months after treatment, menstrual pain days for the moxibustion group was reduced to 1.29±1.06(days) from baseline 5.38±2.79(days). Besides, the improvement in days with menstrual days lasted to six months after randomization for both treatments. But moxibustion is more effective in alleviating menstrual pain-related symptoms, such as emotional symptoms and digestive tract symptom. In this study, we observed that the severity and duration of menstrual symptoms were significantly improved after treatment. The changes in symptoms were significant for either treatment, but the improvement in moxibustion group significantly exceeded the control group since the 5^th^ month for symptom severity and the 4^th^ month for symptom duration(*P*<0.01). Therefore, moxibustion seems to be more effective in alleviating PD-related symptoms, especially from a relatively long-term perspective.

Our study showed that moxibustion may provide long-lasting benefits for PD, especially for improving PD-related symptoms. This may give moxibustion the chance to be incorporated with conventional drugs in long-term management of PD. At present, the nonsteroidal anti-inflammatory drugs, NSAIDs, are frequently prescribed for PD patients in clinics for managing acute pain attacks. The mechanism for NSAIDs to alleviate PD is depending on the inhibition of cyclooxygenase (COX), an enzyme responsible for the formation of prostaglandin (and other prostanoids). The excessive or imbalanced amount of prostanoids (hormone-like substances including prostaglandin) released from the endometrium during menstruation is a major cause of the frequent and dysrhythmic contractions in the uterus with reduced local blood flow and increased sensitization of the peripheral nerves when PD occurs[13, 46]. COX enzymes have two forms, COX-1 and COX-2. Traditional NSAIDs is considered ‘non-selective’ due to its inhibition in both enzyme forms. Study show that it is not effective in 20–25% of patients[47]. However, only COX-2 is responsible for the pathology of PD. The inhibition of COX-1 may lead to many side effects in both gastrointestinal and central-nervous system. Though more ‘targeted’ COX-2 inhibitors, such as meloxicam and MK966, were developed since the 1990s, there are still cardiovascular and/or dermatological adverse events associated with it. Plus its high health economics cost, its clinical use has been narrowed in recent days[48]. On the other hand, the economic costs of moxibustion is increasingly high in most foreign countries. This may considerably hinder the use of moxibustion in clinics. One possible solution is to add moxibustion to drugs to minimize the treatment course. Therefore, in clinical practice, moxibustion could be used as an add-on to more sustainably control menstrual pain. However, the clinical decision should be left to the discretion of patients and their physician.

At present, our knowledge about PD’s pathology has been greatly improved. Many studies indicated that PD was not merely a disease in the uterus, it also related to abnormalities of brain function and structure. PGF_2α_, OT, β-EP, vWF and PGE_2_ are very important in the development of menstrual pain. Studies show that PGs and EP are able to induce contractions of myometrium of the uterus[49]. PGF_2a_ is able to cause potent vasoconstriction of uterine blood vessels and myometrial contraction, both of which decrease blood supply to the uterus. OT as a kind of pituitary neural hormone can stimulate the synthesis and release of PGF_2a_. PGF_2a_ can also increase the release of the OT without affecting its synthesis. This study showed that after treatment the serum level of PGF_2α_, OT, vWF and PGE_2_ were significantly reduced, suggesting that moxibustion might regulate the hypercontractility of the uterus during menstrual pain via regulating the serum levels of pain mediators. The possible mechanisms by which moxibustion works includes temperature-related factors and non-temperature-related factors. The latter includes smoke effects, herbal effects and biophysical effects[50]. Heat from moxibustion may induce vessel dilation to increase the blood flow. In this study, we applied several moxibustion stimulations over several acupoints in the lower abdomen, which are located anatomically above the uterus[51]. The heat stimuli may increase the blood flow in vessels near uterus and consequently result in the dilution of intravascular PGs, bradykinin, histamine. Increased blood flow also improves tissue oxygenation[52]. Other studies have provided explanations about heat applying to the upper abdomen increasing gastrointestinal motility[53]. Other studies show that the physiological effects of thermal therapy were transported via nervous, vascular, and biophysical pathways[54]. Moreover, PD was shown to be associated with significant changes in brain function[55] and structure changes[56, 57]. Functional or structural abnormalities in certain brain regions, such as prefrontal cortex, posterior cingulate cortex etc., were recently found to be of closely associated with pain perception and transmission, higher level sensory processing, and affect regulation, etc. It is possible that moxibustion may exert treatment effect like acupuncture through regulating pain matrix and the reward system in brain[58]. Therefore, the mechanism of moxibustion may extend from regulating target organ function to influence the brain function and structure. More studies are warranted to attest this hypothesis.

Some limitations should be taken into considerations when interpreting the results of this study. First, we only used subjective measurement (VAS and COX menstrual symptom scale) to measure pain condition. More objective measures for pain are warranted. Second, blinded, double-blinded, or triple blinded treatments were not possible in our study since these two treatment strategies are so different. This may undeniably compromise the credibility of the study. Third, most participants of this study were college students. This may limit the generalization of the results to a more diverse patient group. Future study with participants of different backgrounds are needed.

## Conclusions

This study demonstrated that moxibustion therapy is as effective as conventional drug for pain relief in patients with PD. Its therapeutic effect can sustain to 3 months after treatment. Furthermore, moxibustion could be more effective for long-term management of PD-related symptoms. Therefore, both treatment strategies showed desirable merits in managing menstrual pain, given their treatment effects and economic costs. However, this study as a pragmatic trial only demonstrates the effectiveness, not the efficacy, of moxibustion for menstrual pain. It didn’t rule out the effect of psychological factors during treatment process, because no blind procedure or sham control was used due to availability. In clinical practice, moxibustion should be used at the discretion of patients and their physicians.

## Supporting information

S1 Data Individual patient data of the experimental group. (XLS)

S2 Data Individual patient data of the control group. (XLS)

S3 Data Data of lab tests for blood sample. (XLS)

S1 File Ethical approval notice. Ethical approval from Sichuan Regional Ethics Review Committee on Traditional Chinese Medicine (2013KL-004).
(JPG)

S2 File Study protocol. Published study protocol.
(PDF)

S3 File PPA results. Results of per-protocol analysis.
(DOCX)

S4 File Consort 2010 checklist. The results are reported in accordance with the CONSORT guideline.
(DOC)

S5 File STRICTA checklist. The reporting of acupuncture/moxibustion treatment is based on STRICTA recommendations.
(DOCX)

## References

[1] Kennedy S. Primary dysmenorrhoea. The Lancet. 1997;349(9059):1116. 10.1016/S0140-6736(05)63018-8 9113008

[2] Grandi G, Ferrari S, Xholli A, Cannoletta M, Palma F, Romani C, et al Prevalence of menstrual pain in young women: what is dysmenorrhea? Journal of pain research. 2012;5:169 10.2147/JPR.S30602 22792003 PMC3392715

[3] Zahradnik H-P, Hanjalic-Beck A, Groth K. Nonsteroidal anti-inflammatory drugs and hormonal contraceptives for pain relief from dysmenorrhea: a review. Contraception. 2010;81(3):185–96. 10.1016/j.contraception.2009.09.014 20159173

[4] Harel Z. Dysmenorrhea in adolescents. Annals of the New York Academy of Sciences. 2008;1135(1):185–95. 18574224 10.1196/annals.1429.007

[5] Dawood M. Dysmenorrhea. The Journal of reproductive medicine. 1985;30(3):154 3158737

[6] Yang J, Chen J, Lao L, Yang M, Chen J, Bo L, et al Effectiveness study of moxibustion on pain relief in primary dysmenorrhea: study protocol of a randomized controlled trial. Evidence-Based Complementary and Alternative Medicine. 2014;2014. 10.1155/2014/434978 PMC3996889 24803947

[7] Ma YX, Ma LX, Liu Xl, Ma YX, Lv K, Wang D, et al A Comparative Study on the Immediate Effects of Electroacupuncture at Sanyinjiao (SP6), Xuanzhong (GB39) and a Non‐Meridian Point, on Menstrual Pain and Uterine Arterial Blood Flow, in Primary Dysmenorrhea Patients. Pain Medicine. 2010;11(10):1564–75. 10.1111/j.1526-4637.2010.00949.x 21199306

[8] Wang M-C, Hsu M-C, Chien L-W, Kao C-H, Liu C-F. Effects of auricular acupressure on menstrual symptoms and nitric oxide for women with primary dysmenorrhea. The Journal of Alternative and Complementary Medicine. 2009;15(3):235–42. 10.1089/acm.2008.0164 19292653

[9] Shi G-X, Liu C-Z, Zhu J, Guan L-P, Wang D-J, Wu M-M. Effects of acupuncture at Sanyinjiao (SP6) on prostaglandin levels in primary dysmenorrhea patients. The Clinical journal of pain. 2011;27(3):258–61. 10.1097/AJP.0b013e3181fb27ae 21358291

[10] Ortiz MI. Primary dysmenorrhea among Mexican university students: prevalence, impact and treatment. European Journal of Obstetrics & Gynecology and Reproductive Biology. 2010;152(1):73–7. 20478651 10.1016/j.ejogrb.2010.04.015

[11] Unsal A, Ayranci U, Tozun M, Arslan G, Calik E. Prevalence of dysmenorrhea and its effect on quality of life among a group of female university students. Upsala journal of medical sciences. 2010;115(2):138–45. 10.3109/03009730903457218 20074018 PMC2853792

[12] French L. Dysmenorrhea. American family physician. 2005;71(2):285 15686299

[13] Dawood MY. Primary dysmenorrhea: advances in pathogenesis and management. Obstetrics & Gynecology. 2006;108(2):428–41. 16880317 10.1097/01.AOG.0000230214.26638.0c

[14] Zorbas KA, Economopoulos KP, Vlahos NF. Continuous versus cyclic oral contraceptives for the treatment of endometriosis: a systematic review. Archives of gynecology and obstetrics. 2015;292(1):37–43. Epub 2015/02/04. 10.1007/s00404-015-3641-1 25644508

[15] Koga K, Takamura M, Fujii T, Osuga Y. Prevention of the recurrence of symptom and lesions after conservative surgery for endometriosis. Fertility and sterility. 2015;104(4):793–801. Epub 2015/09/12. 10.1016/j.fertnstert.2015.08.026 26354093

[16] Proctor M, Murphy PA. Herbal and dietary therapies for primary and secondary dysmenorrhoea. The Cochrane Library. 2001. 10.1002/14651858.CD002124 11687013

[17] Yang J, Yu S, Lao L, Yang M, Chen J, Luo X, et al Use of moxibustion to treat primary dysmenorrhea at two interventional times: study protocol for a randomized controlled trial. Trials. 2015;16(1):35. 25633584 10.1186/s13063-015-0552-1 PMC4347976

[18] Kobayashi K. Induction of heat-shock protein (hsp) by moxibustion. The American journal of Chinese medicine. 1995;23(03n04):327–30. 8571930 10.1142/S0192415X95000390

[19] Chang X-R, Peng L, Yi S-X, Peng Y, Yan J. Association of high expression in rat gastric mucosal heat shock protein 70 induced by moxibustion pretreatment with protection against stress injury. World journal of gastroenterology: WJG. 2007;13(32):4355–9. 10.3748/wjg.v13.i32.4355 17708611 PMC4250864

[20] Kawakita K, Shinbara H, Imai K, Fukuda F, Yano T, Kuriyama K. How do acupuncture and moxibustion act?-Focusing on the progress in Japanese acupuncture research. Journal of pharmacological sciences. 2006;100(5):443–59. 16799260 10.1254/jphs.crj06004x

[21] Okada K, Kawakita K. Analgesic action of acupuncture and moxibustion: a review of unique approaches in Japan. Evidence-Based Complementary and Alternative Medicine. 2009;6(1):11–7. 10.1093/ecam/nem090 18955231 PMC2644273

[22] Kim J-I, Choi J-Y, Lee H, Lee MS, Ernst E. Moxibustion for hypertension: a systematic review. BMC cardiovascular disorders. 2010;10(1):33. 20602794 10.1186/1471-2261-10-33 PMC2912786

[23] Tian-Hua W. Effects of moxibustion or acupoint therapy for the treatment of primary dysmenorrhea: a meta-analysis. Alternative therapies in health and medicine. 2014;20(4):33 25141361

[24] Chung Y-C, Chen H-H, Yeh M-L. Acupoint stimulation intervention for people with primary dysmenorrhea: systematic review and meta-analysis of randomized trials. Complementary therapies in medicine. 2012;20(5):353–63. 10.1016/j.ctim.2012.02.008 22863651

[25] Kim JW, Park BK, Jeon JI, Yim YK. Acupuncture and Moxibustion for Primary Dysmenorrhea in Korean Literatures: A Systematic Review of Randomized Controlled Trials. The Acupuncture. 2015;32(2):123–30.

[26] Lefebvre G, Pinsonneault O, Antao V, Black A, Burnett M, Feldman K, et al Primary dysmenorrhea consensus guideline. J Obstet Gynaecol Can. 2005;27(12):1117–46. 16524531 10.1016/s1701-2163(16)30395-4

[27] Yu S, Yang J, Yang M, Gao Y, Chen J, Ren Y, et al Application of Acupoints and Meridians for the Treatment of Primary Dysmenorrhea: A Data Mining-Based Literature Study. Evidence-Based Complementary and Alternative Medicine. 2015;2015. 10.1155/2015/752194 PMC4354715 25802545

[28] Bu Y, Chen S, Du G. Study on the modern application of acupoints for primary dysmenorrhea. Journal of Traditional Chinese Medicine. 2010;9:811–9.

[29] Smith CA, Crowther CA, Petrucco O, Beilby J, Dent H. Acupuncture to treat primary dysmenorrhea in women: A randomized controlled trial. Evidence-Based Complementary and Alternative Medicine. 2011;2011. 10.1093/ecam/nep239 PMC3140031 21799683

[30] Ju H, Jones M, Mishra G. The prevalence and risk factors of dysmenorrhea. Epidemiol Rev. 2014;36(1):104–13. Epub 2013/11/29. 24284871 10.1093/epirev/mxt009

[31] Ayan M, Sogut E, Tas U, Erdemir F, Sahin M, Suren M, et al Pain levels associated with renal colic and primary dysmenorrhea: a prospective controlled study with objective and subjective outcomes. Archives of gynecology and obstetrics. 2012;286(2):403–9. 10.1007/s00404-012-2316-4 22476379

[32] Rigi SN, Navidian A, Safabakhsh L, Safarzadeh A, Khazaian S, Shafie S, et al Comparing the analgesic effect of heat patch containing iron chip and ibuprofen for primary dysmenorrhea: a randomized controlled trial. BMC women's health. 2012;12(1):25. 22913409 10.1186/1472-6874-12-25 PMC3492023

[33] Harel Z. Dysmenorrhea in adolescents and young adults: an update on pharmacological treatments and management strategies. Expert opinion on pharmacotherapy. 2012;13(15):2157–70. 10.1517/14656566.2012.725045 22984937

[34] Glickman-Simon R, Lindsay T. Cannabinoids for Chronic Pain, Mediterranean Diet and Cognitive Function; Vitamin E and Selenium for Cataract Prevention; Acupuncture and Moxibustion for Primary Dysmenorrhea; Massage Therapy and In Vitro Fertilization. Explore: The Journal of Science and Healing. 2015;11(6):489–93. 10.1016/j.explore.2015.08.013 26525669

[35] Cho SH, Hwang EW. Acupuncture for primary dysmenorrhoea: a systematic review. BJOG: An International Journal of Obstetrics & Gynaecology. 2010;117(5):509–21. 20184568 10.1111/j.1471-0528.2010.02489.x

[36] Langevin HM, Schnyer R, MacPherson H, Davis R, Harris RE, Napadow V, et al Manual and electrical needle stimulation in acupuncture research: pitfalls and challenges of heterogeneity. The Journal of Alternative and Complementary Medicine. 2015;21(3):113–28. 10.1089/acm.2014.0186 25710206 PMC4855731

[37] Ma Y-x, Yang X-y, Guo G, Du D-q, Yu Y-p, Gao S-z. Research of Herb-Partitioned Moxibustion for Primary Dysmenorrhea Patients Based on the LC-MS Metabonomics. Evidence-Based Complementary and Alternative Medicine. 2015;501:621490. 10.1155/2015/621490 PMC4502312 26229545

[38] Lee IS. Effect of moxibustion heat therapy on menstrual cramps, dysmenorrhea, and activities of daily living. Journal of Korean Public Health Nursing. 2004;18(1):39–49.

[39] Gao J, Wang Q, Xian S, Feng Y-m, Cao W-x, Ye J-y, et al The effect of moxibustion on alleviating menstrual pain in a population of young nursing students: A prospective randomized cross-over pilot study. Complementary Therapies in Medicine. 2015;23(6):773–81. 10.1016/j.ctim.2015.08.005 26645515

[40] Sun L, Ge J, Yang J, She Y, Li W, Li X, et al [Randomized controlled clinical study on ginger-partitioned moxibustion for patients with cold-damp stagnation type primary dysmenorrhea]. Zhen ci yan jiu = Acupuncture research/[Zhongguo yi xue ke xue yuan Yi xue qing bao yan jiu suo bian ji]. 2009;34(6):398–402. 20209976

[41] Xue Z, Liu C, Gao S, Ma Y. [The herbal-partitioned moxibustion for primary dysmenorrhea and it's impact on reproductive endocrinal function of patients]. Zhongguo zhen jiu = Chinese acupuncture & moxibustion. 2014;34(3):209–12. 24843954

[42] Zhu Y, Chen R, Le J, Miao F. [Efficacy observation of primary dysmenorrhea treated with isolated-herbal moxibustion on Shenque (CV 8)]. Zhongguo zhen jiu = Chinese acupuncture & moxibustion. 2010;30(6):453–5. 20578380

[43] Helms PJ. ‘Real world’pragmatic clinical trials: what are they and what do they tell us? Pediatric allergy and immunology. 2002;13(1):4–9. 12000492 10.1034/j.1399-3038.2002.00194.x

[44] Chalkidou K, Tunis S, Whicher D, Fowler R, Zwarenstein M. The role for pragmatic randomized controlled trials (pRCTs) in comparative effectiveness research. Clinical trials. 2012;9(4):436–46. 10.1177/1740774512450097 22752634

[45] Luce BR, Kramer JM, Goodman SN, Connor JT, Tunis S, Whicher D, et al Rethinking randomized clinical trials for comparative effectiveness research: the need for transformational change. Annals of Internal Medicine. 2009;151(3):206–9. 19567619 10.7326/0003-4819-151-3-200908040-00126

[46] Dawood MY, Khan-Dawood FS. Clinical efficacy and differential inhibition of menstrual fluid prostaglandin F 2α in a randomized, double-blind, crossover treatment with placebo, acetaminophen, and ibuprofen in primary dysmenorrhea. American journal of obstetrics and gynecology. 2007;196(1):35.e1–.e5. 17240224 10.1016/j.ajog.2006.06.091

[47] Modaress Nejad V, Asadipour M. Comparison of the effectiveness of fennel and mefenamic acid on pain intensity in dysmenorrhoea. 2006. 17037712

[48] Marjoribanks J, Ayeleke RO, Farquhar C, Proctor M. Nonsteroidal anti-inflammatory drugs for dysmenorrhoea. status and date: New search for studies and content updated (conclusions changed), published in. 2015;(7). 10.1002/14651858.CD001751.pub3 PMC6953236 26224322

[49] Arulkumaran S. The roles of prostanoid EP receptors in the control of contractions of human myometrium: Imperial College London; 2012.

[50] Chiu J-H. How does moxibustion possibly work? Evidence-Based Complementary and Alternative Medicine. 2013;2013.

[51] Deadman P, Al-Khafaji M, Baker K. A manual of acupuncture: Journal of Chinese Medicine Publications East Sussex, UK; 1998.

[52] Steen M, Cooper K. Cold therapy and perineal wounds: too cool or not too cool? British Journal of Midwifery. 1998;6(9):572–9.

[53] Akin MD, Weingand KW, Hengehold DA, Goodale MB, Hinkle RT, Smith RP. Continuous low-level topical heat in the treatment of dysmenorrhea. Obstetrics & Gynecology. 2001;97(3):343–9. 11239634 10.1016/s0029-7844(00)01163-7

[54] Reading AE. A comparison of the McGill Pain Questionnaire in chronic and acute pain. Pain. 1982;13(2):185–92. 6889722 10.1016/0304-3959(82)90028-8

[55] Wei S-Y, Chao H-T, Tu C-H, Li W-C, Low I, Chuang C-Y, et al Changes in functional connectivity of pain modulatory systems in women with primary dysmenorrhea. Pain. 2016;157(1):92–102. 10.1097/j.pain.0000000000000340 26307856

[56] Tu C-H, Niddam DM, Chao H-T, Chen L-F, Chen Y-S, Wu Y-T, et al Brain morphological changes associated with cyclic menstrual pain. PAIN. 2010;150(3):462–8. 10.1016/j.pain.2010.05.026 20705214

[57] Tu C-H, Niddam DM, Chao H-T, Liu R-S, Hwang R-J, Yeh T-C, et al Abnormal cerebral metabolism during menstrual pain in primary dysmenorrhea. Neuroimage. 2009;47(1):28–35. 10.1016/j.neuroimage.2009.03.080 19362153

[58] Lee I-S, Wallraven C, Kong J, Chang D-S, Lee H, Park H-J, et al When pain is not only pain: Inserting needles into the body evokes distinct reward-related brain responses in the context of a treatment. Physiology & behavior. 2015;140:148–55. 25528104 10.1016/j.physbeh.2014.12.030

