# Supplementary figures and images for: Moxibustion for pain relief in patients with primary dysmenorrhea: A randomized controlled trial
Source: PLoS One. 2017 Feb 7;12(2):e0170952. doi: 10.1371/journal.pone.0170952 (PMC5295763; doi:10.1371/journal.pone.0170952)

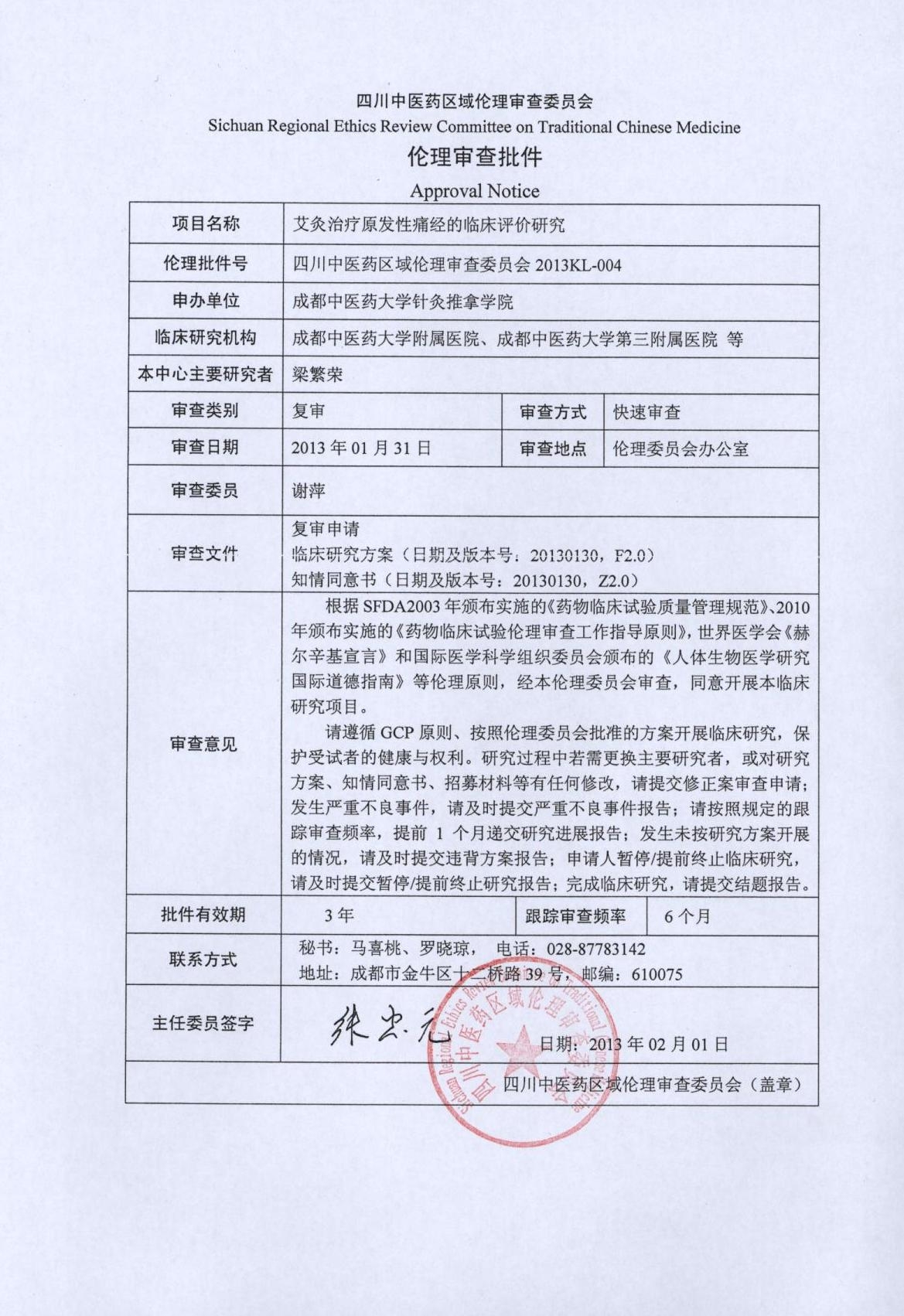

Supplement: S1 File — Ethical approval from Sichuan Regional Ethics Review Committee on Traditional Chinese Medicine (2013KL-004). (JPG) [file pone.0170952.s004.jpg]
