# Supplementary material for: Moxibustion for pain relief in patients with primary dysmenorrhea: A randomized controlled trial
Source: PLoS One. 2017 Feb 7;12(2):e0170952. doi: 10.1371/journal.pone.0170952 (PMC5295763; doi:10.1371/journal.pone.0170952)

Hindawi Publishing Corporation
Evidence-Based Complementary and Alternative Medicine
Volume 2014, Article ID 434978, 6 pages
http://dx.doi.org/10.1155/2014/434978

Research Article

Effectiveness Study of Moxibustion on Pain Relief in Primary Dysmenorrhea: Study Protocol of a Randomized Controlled Trial

Jie Yang,[1,2] Jiao Chen,[1] Lixing Lao,[2] Mingxiao Yang,[1] JianPing Chen,[1,2] Linna Bo,[1] Hongzhi Tang,[1] Ling Yi,[3] Hui Zheng,[1] Xi Wu,[1] and Fanrong Liang[1]

[1] *Chengdu University of Traditional Chinese Medicine, Chengdu, Sichuan 610072, China*
[2] *School of Chinese Medicine, The University of Hong Kong, Hong Kong*
[3] *Medical Center and Hospital of Qionglai, Qionglai, Sichuan 611530, China*

Correspondence should be addressed to Xi Wu; wuxi403@hotmail.com and Fanrong Liang; acuresearch@126.com

Received 24 November 2013; Revised 22 February 2014; Accepted 2 March 2014; Published 2 April 2014

Academic Editor: Helen G. Hall

Copyright © 2014 Jie Yang et al. This is an open access article distributed under the Creative Commons Attribution License, which permits unrestricted use, distribution, and reproduction in any medium, provided the original work is properly cited.

Dysmenorrhea is a prevalent problem in menstruating women. As a nonpharmacologic and free of relevant side effects intervention, moxibustion is considered as a safe treatment and has long been recommended for dysmenorrhea in China. However, the exact effects of moxibustion in PD have not been fully understood. Therefore we designed this random clinical trial aiming to (1) investigate whether moxibustion is safe and effective for pain relief in primary dysmenorrhea when compared to conventional pain-killers and (2) assess the acceptability and side effects associated with moxibustion. The results of this trial will contribute to a better understanding of the different effects of moxibustion in pain relief in primary dysmenorrhea when compared to conventional pharmacologic pain treatment.

1. Background

Dysmenorrhea is the leading cause of recurrent short-term school absenteeism among adolescent girls and a prevalent problem in menstruating women [1]. It usually occurs before or during menstruation, emanating from lower abdominal or pelvic pain and radiating to the back and inner thighs. It may be a dull, throbbing, or spasmodic but always cyclical period pain [2]. According to Women's Health Specialist Library in 2009, dysmenorrhea can be divided into two broad categories: primary and secondary dysmenorrhea. Primary dysmenorrhea (PD) usually dates from the onset of ovulatory cycles without any obvious underlying disease. While in secondary dysmenorrhea, there is usually gross pathology in the pelvic structure [3]. Primary dysmenorrhea typically begins before and is relieved soon after the onset of menstruation. There is considerable variation in the prevalence of dysmenorrhea, depending on the definition used: 45–72% of all women and 43–93% of adolescent girls experience dysmenorrhea [2]. In contrast to PD, the pain in secondary dysmenorrhea is usually relieved after the correction in pelvic abnormalities. At present, nonsteroidal anti-inflammatory medication is the main treatment for PD, which is accompanied with oral contraceptive pill (COCP) when necessary [4], though with a number of side effects [5]. As a result, about 80% and 30% of adult women used nonprescription and over-the-counter drugs, respectively, for dysmenorrhea because they believed that conventional medications were not effective or had unpleasant effects, or simply because they do not like taking any medications [6].

As a nonpharmacologic intervention, moxibustion is recommended as a safe option comparing to conventional drug treatment and has long been applied in dysmenorrhea in China [7]. Various methods of moxibustion have been applied in clinical therapies, generally including moxibustion with moxa cones, moxa rolls, and other apparatus. Moxibustion with moxa cones can be divided into direct and indirect manners, which are differentiated by whether or not the moxa is adhered to the skin without media. As the most traditional and widely used method, moxibustion

with moxa (i.e., *Artemisia vulgaris* or mugwort) places an ignited mugwort directly or indirectly at the acupoints to generate heat at various temperatures to subsequently stimulate and activate the functions of acupoints. The possible underlying mechanisms of moxibustion may be conducted through temperature-related and non-temperature-related factors. According to a recent review, the heat stimulation of moxibustion can activate inflammatory responses and induce vascular changes [8]. As demonstrated by animal experiments, local dermal mediators such as histamine and substance P are released to induce vasodilatation in mice [9]. Moreover, it is concluded that the effects of moxibustion are composed of smoke effects, herbal effects, and biophysical effects (far infrared). Though many studies have proposed plausible mechanisms, a recent systematic review on clinical trials assessing the effects of acupuncture-related therapies (including moxibustion) indicated no convincing outcomes after these treatments for PD [10]. Hence, there is an urgent need for clinical trials with sound methodological design to assess the effects of moxibustion.

Therefore we designed this random clinical trial aiming to (1) investigate whether moxibustion is safe and effective for pain relief in primary dysmenorrhea when compared to conventional pain-killers and (2) assess the acceptability and side effects associated with moxibustion.

2. Methods

2.1. Design. This trial is a randomized, open-labeled, drug-controlled clinical trial comprising two parallel arms, which are moxibustion and drug treatment, respectively. The study for each participant lasts for nine menstrual cycles, spending 3 menstrual cycles in baseline, treatment, and follow-up periods, respectively. Figure 1 showed the flow chart of this trial. This study will be conceived under the guidance and principles for clinical trials (including the Declaration of Helsinki, the International Conference on Harmonization (ICH), and WHO Good Clinical Practice standard). Sichuan Regional Ethics Review Committee on Traditional Chinese Medicine has approved all research procedures (2013KL-004) and has been registered in Clinicaltrials.gov, National Institute of Health, USA [11] (NCT01972906).

2.2. Participants. This trial will be carried out in Chengdu University of Traditional Chinese Medicine, Chengdu, Sichuan, China. All participants included in this trial should be diagnosed with primary dysmenorrhea in the outpatient Departments of General Practice, Acupuncture, and Gynecology in the 3rd Teaching Hospital of Chengdu University of TCM. A total amount of 152 patients diagnosed with primary dysmenorrhea in accordance with the Clinical Guideline of Primary Dysmenorrhea by the Society of Obstetricians and Gynaecologists of Canada will be considered as eligible patients. Then they will be briefly introduced to this study and asked to fill in a baseline questionnaire at the initial visit to their gynecology doctors. According to the baseline information, eligible participants will be informed about all the benefits as well as potential risks that they may encounter in this trial, and they are free to withdraw the study at any time without any specific reason. Further procedures such as recruitment, regular physical examination, and randomization will be proceeded if the patients provide a hard copy of agreement. Study period of this study was expected to be from February 2012 to June 2014.

2.3. Inclusion Criteria. Eligible participants should meet the following inclusion criteria: (1) being aged from 13 to 35 years with a history of regular menstrual cycles (28 days ± 7 days); (2) having experienced menstrual pain of intensity from moderate to severe and the visual analog scale (VAS) ≥40 mm for at least 3 menstrual cycles before this study; (3) the syndrome differentiation of traditional Chinese medicine correlating Qi-stagnation and blood stasis syndrome and congealing cold-damp syndrome; and (4) providing a hard-copy of agreement form.

2.4. Exclusion Criteria. Participants with any of the following conditions will be excluded: (1) women with secondary dysmenorrhea caused by endometriosis, pelvic inflammation, or myomas of uterus confirmed by type-B ultrasound exam by gynecologists; (2) women with irregular menstrual cycles; (3) women with uncontrolled diagnosed neurological diseases, immunodeficiency, bleeding disorders, and allergies; (4) women with uncontrolled medical conditions which are unfit for moxibustion; (5) women taking prostaglandin synthetase inhibitor (PGSI) two weeks before inclusion; (6) women in lactation, pregnant women, or those with plans to get pregnant in the coming half year; (7) women taking drugs such as NSAIDs or oral contraceptive pills that can affect the outcomes; (8) women receiving moxibustion currently or received moxibustion 2 weeks prior to enrollment; and (9) women undergoing other trials.

2.5. Randomization. After physical examination at the second visit to the doctor following the enrollment, a patient coordinator collects and inputs each patient's medical history and baseline information into an Excel file with her own account. A data processor that has not accessed the random information will separate the demographic details from baseline information. The demographic information will be sent to a third researcher who is not involved in data collection and disease diagnosis, to further randomly allocate the patients. A random digit table will be used for assigning the participants equally into two groups using two blocks. Odd numbers will be assigned to the patients in one block and even numbers to the patients in the other block. The randomization code is available only to the third researcher who has not participated in the patient recruitment and treatment. The numbered sealed opaque envelope will be used to keep the randomization code and will not be disclosed to other researchers until the statistical analysis has been completed by researchers. All participants will be assessed and the results will be analyzed by professional researchers blinded in respect of patient allocation and treatments.

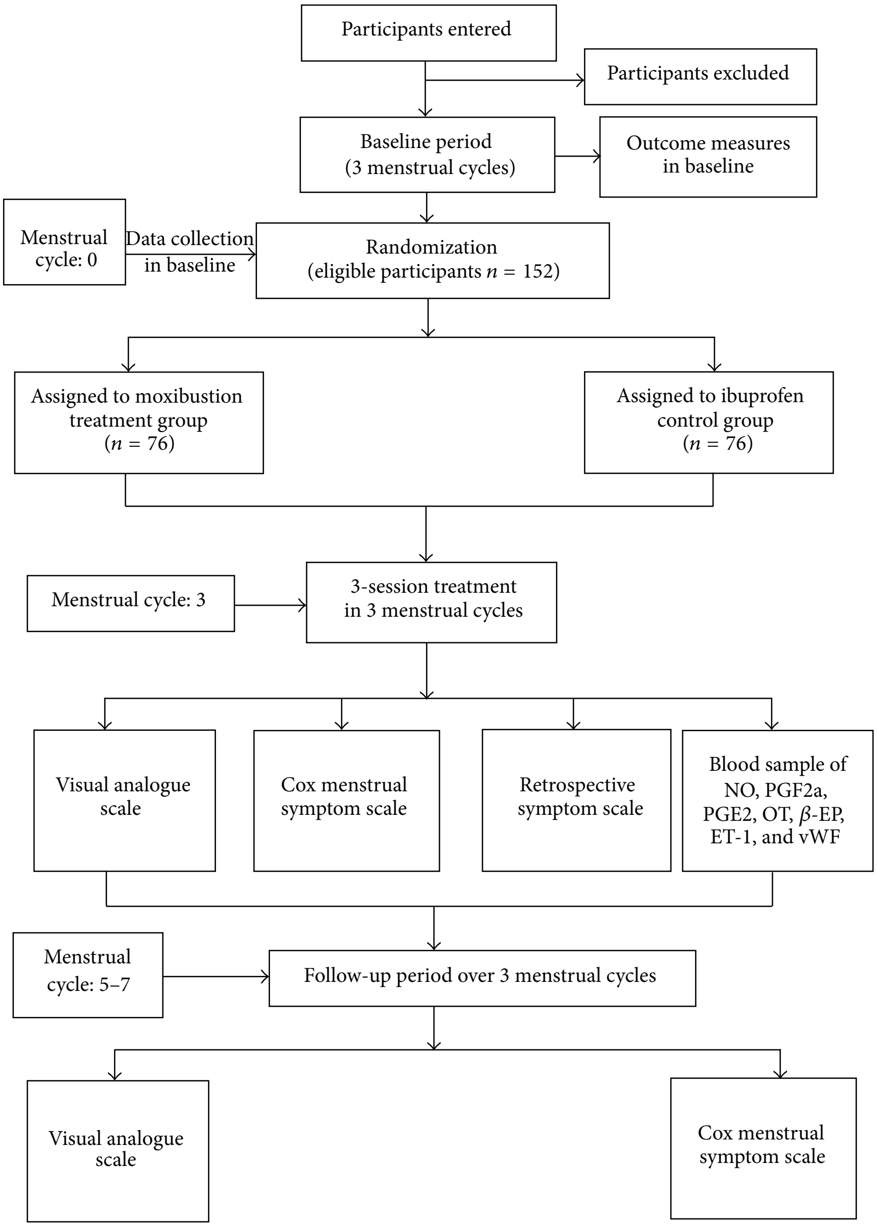

Figure 1: Trial flow chart.

3. Blinding

Because this trial compares the effects of moxibustion with conventional analgesics, it is unable to blind the patients and practitioners. Thus, the patients know which group they are in, and they will be informed that the two kinds of treatments are useful for primary dysmenorrhea. However, data collectors and statisticians will be blinded to the setting and treatments of different groups.

3.1. Interventions. Patients will receive different interventions depending on the groups they are assigned to. For the conventional control group, patients will be instructed to administrate ibuprofen sustained release capsules (0.3 g/capsule × 12 capsules, Sino-GlaxoSmithKline, Tianjin, China). For the moxibustion group, two different TCM patterns of acupoints are selected for treatments. The rationale for this acupoint selection strategy is based on a TCM perspective that those two kinds of acupoint combination are all useful and effective in the management of primary dysmenorrhea. The syndromes and the associated symptoms for differentiating will be drawn from the Traditional Chinese Medicine Professional Statute: Criteria of Diagnosis and Therapeutic Effect of Diseases/Syndromes, published by the State of Administration of TCM, PRC. Two main diagnostic syndromes defined and used in this study are listed in Table 1 [12]. Treatment details are listed as follows.

Table 1: TCM pattern differentiation protocol for women with primary dysmenorrhoea.

Diagnostic pattern 1	Items
Qi-stagnation and blood stasis	Pain: distended, stabbed abdominal pain before or during the period aggravated by pressure.<br>Menstruation: dark purplish in colour, clotty, and hesitant or scanty in flow; pain relieved after clots discharged.<br>Accompanying symptoms: distending in breasts, mood swings.<br>Tongue: purplish.<br>Pulse: wiry.
Diagnostic pattern 2	Items
Congealing cold-damp	Pain: distended, stabbed abdominal pain before or during the period, favourably to warmth, lower back pain.<br>Menstruation: dark purplish in colour, clotty, and hesitant in flow; pain relieved after clots discharged.<br>Accompanying symptoms: distending in breasts, mood swings, and aversion to cold.<br>Tongue: pale and white greasy tongue coating.<br>Pulse: deep and slow.

3.1.1. Moxibustion Treatment Group. The design of the moxibustion intervention and the point prescription are based on the theory of TCM as well as the literature. It is demonstrated that Guanyuan (CV4), Shenque (CV8), and Sanyinjiao (SP6) are the most frequently used acupoints [13]. Thus they are selected as key acupoints receiving moxa heat stimulation. The location and manipulations of acupoints in the moxibustion treatment group are shown in Table 2.

Mild moxibustion including moxibustion without dermal contact and moxa roll will be used, which is made of mugwort with a paper cylinder (Z32021062, Oriental Moxa Co., Suzhou, China). The participant was asked to perform the treatment in a comfortable supine position and the skin surface at every point was sterilized. The top of the moxa roll is adequately ignited and then applied approximately 2-3 cm above the dermal layer of acupoints. Moxibustion treatment was conducted on the acupoints CV4 and CV6 at the same time. Right after that the SP6 at both sides of the body were moxibusted simultaneously. The intensity of moxibustion is increased to induce a mild warm and comfortable sensation to the patient before the skin is provoked with hyperaemia. Moxibustion at each point commonly lasts for about 10 to 15 minutes. The entire treatment process for each patient lasts for about 25 to 30 minutes. Moxibustion treatment will start 7 days before the beginning of menses until forthcoming menstruation. Participants in this group will receive moxibustion treatment once a day, 7 days a session for a total of 3 sessions over 3 menstrual cycles. After 3-session treatment, there will be a 3-menstrual cycle follow-up period.

Moxibustion practitioner in this trial had over 6 years' experience in acupuncture moxibustion and Traditional Chinese Medicine (TCM) training and at least 2 years of clinical experience in an academic acupuncture and moxibustion clinic. The treatment points and moxibustion procedures were selected in a consensus process by professors and researchers in acupuncture and moxibustion.

3.1.2. Ibuprofen Sustained Release Capsules Control Group. All the participants in this group will be instructed to use ibuprofen sustained release capsules for pain relief. They will receive 0.3 g capsules per time, twice a day for three menstrual cycles. Every session is one day before every menstrual cycle, lasting three days. A further followup will last for three menstruation cycles.

During the trial, all participants should not take any acute analgesic medication for dysmenorrhea pain except that given by the investigator. However, the participant will be permitted to use the necessary analgesics such as indometacin, if she cannot endure the pain. The quantity and time of pain killer pills taken during the menstrual period will be documented in the case-report form (CRF) at every visit.

3.2. Outcome Measurement. The effectiveness of moxibustion for pain relief on PD was assessed by the primary outcome measure: change from baseline in menstrual pain intensity measured by VAS at 6 menstrual cycles (at baseline and at 1st, 2nd, 3rd, 4th, 5th, and 6th menstrual cycle after inclusion). The VAS is a tool widely used to measure pain. Participants will be asked to indicate a perception of pain intensity (most commonly) scored from 1 to 10 (0, no pain; 10, maximum) along a 100 mm horizontal line.

The secondary outcome measures are (1) the Cox menstrual symptom scale; (2) the retrospective symptom scale; and (3) blood sample of NO, PGF2a, PGE2, OT, β-EP, ET-1, and vWF. Assessments of NO, PGF2a, PGE2, OT, β-EP, ET-1, and vWF will be performed at baseline and at the fourth menstrual cycle after inclusion (after completion of three sessions of treatment).

4. Safety

Any adverse event such as pain, scald, or faint or severe adverse event that has been experienced by the patient in the treatment process should be reported to the researcher and, moreover, should be carefully recorded in the case-report form.

Table 2: Details of acupoints in moxibustion treatment group [14].

Group	Acupoints	Location	Gynecological indication
Moxibustion treatment group	(1) Guanyuan (CV4)	(1) On the midline of the lower abdomen, 3 cun inferior to the umbilicus and 2 cun superior to the pubic symphysis.	Cold Qi entering the lower abdomen giving rise to pain, cold accumulation with deficiency, running piglet Qi rising to the heart, fullness of the lower abdomen, back pain, and twisting pain below the umbilicus that gradually radiates to the genitals, sudden painful shan disorder, and intense heat in the hypogastrium.
	(2) Shenque (CV8)	(2) In the centre of the umbilicus.	Deficiency coldness of the abdomen, incessant diarrhoea, borborygmus, diarrhoea in the elderly or in deficient people, infantile diarrhoea following breast-feeding, prolapse of the rectum, sudden turmoil disorder, pain around the umbilicus, oedema, and drum distention.
	(3) Sanyinjiao (SP6)	(3) On the medial side of the lower leg, 3 cun superior to the prominence of the medial malleolus, in a depression close to the medial crest of the tibia.	Irregular menstruation, uterine bleeding, uterine bleeding with dizziness, menorrhagia, amenorrhoea, dysmenorrhoea, abdominal (zheng jia) masses in women, leucorrhoea, and uterine prolapse.

5. Sample Size Calculation

Sample size was calculated by a G∗Power 3, developed by Institute for Experimental Psychology, Heinrich-Heine-University, Germany. For this trial, it has been determined prospectively that $\alpha = 0.05$ and $1 - \beta = 0.90$. According to a previous trial on moxibustion for PD [15], a minimum difference of clinical effectiveness is 2 cm in pain relief weighed by the visual analogue scale. Thus, a total of 152 participants will be included in this trial for a compensation to 15% dropout rate, with 76 patients in each group.

6. Data Analysis

Demographic and baseline characteristics of study participants will be elucidated by descriptive statistics. Between-group differences will be tested by using Repeated Measure Analyses of Variance. The accepted level of significance for all analyses was $P < 0.05$. The whole data analysis process will be performed by statisticians who are independent from the research team and blinded to the group settings. The SPSS software (SPSS 12.0 KO for Windows) was used to perform the data analysis.

7. Discussion

This trial highlights assessing the effectiveness of moxibustion therapy versus conventional NSAIDs drugs in pain relief for primary dysmenorrhea. According to the doctrine of traditional Chinese medicine, moxibustion with a warmth and tonic property originated in the application of fire and has been widely applied to manage disorders caused by coldness, dampness, stagnation, and deficiency, all of which are common pathological factors according to TCM theory [16]. In the long history of TCM usage in China, moxibustion as well as acupuncture has long ago been adopted and now utilized to manage various gynecological diseases [17], such as menopausal hot flashes [18], complications after hysterectomy [19], and polycystic ovary syndrome [20]. Primary dysmenorrhea as one of these most common reasons for gynecological visits is believed to benefit from moxibustion by lots of female patients [21]. Nevertheless, as concluded by a recent systematic review, there is no convincing evidence about the effectiveness of moxibustion for lack of good evidence in previous trials, and more trials with RCT (randomized controlled trial) design are needed in the future [10]. As widely acknowledged, the RCT is long regarded as the "gold standard" in clinical research. It is an advantageous tool to assess a specific intervention's effectiveness by avoiding biases resulting from confounding factors such as no randomization and inadequate concealment. Given the current condition, we hence design this trial as a RCT to evaluate the effectiveness of moxibustion for PD in terms of its pain relief in comparison with positive conventional drugs.

As for the outcomes we selected, because VAS is commonly used in the measurement of pain conditions, it can forwardly and accurately manifest the pain condition for multiple patients in multiple measure time [22]. However, its limitation merely to severity of pain makes it insufficient to measure and reflex the complications and durations of symptoms. For this consideration, in this trial the Cox menstrual symptom scale was introduced to be another major outcome measure.

The Cox menstrual symptom scale or CMSS is developed by Professor Daniel J. Cox in 1978; it is frequently used in the assessment of the duration and severity of dysmenorrheal pain. This scale included 18 symptoms in dysmenorrhea, such as lower abdominal pain, nausea, vomiting, poor appetite, lumber pain, leg pain, fatigue, vertigo, diarrhea, change of complexion, gastric pain, insomnia, general pain, depression, and irritation [23]. As shown previously, it covers a wide range of dysmenorrheal symptoms and lots of complications. It can be used to measure the duration and severity of dysmenorrhea more properly. Therefore we adopted it in hope that the option of CMSS may supplement the VAS to comprehensively exhibit the importance of moxibustion for PD in comparison with conventional pain killers.

Meanwhile, as the above 2 measures are generally subjective and are prone to be influenced by lots of irrelevant factors, the effectiveness assessment could not be attested appropriately. Hence, several objective experimental measures are observed as complementary. This trial collected blood sample of participants in 48 hours of menses, that is to say, the venous blood samples were collected when the PGs were at their peak in endometrium [24]. This was thought to be a reasonable approach to objectify the results. Moreover, it may act as a supplement to the primary measurements.

In summarization, it is our best hope that this trial with relatively rigid methodology design in terms of its randomization, control, and blinding to assessor may consequently provide good evidence to elaborate the analgesic effect of moxibustion for primary dysmenorrhea.

Conflict of Interests

The authors declared no conflict of interests.

Authors' Contribution

Jie Yang and Jiao Chen contributed equally to this trial. Jiao Chen, Xi Wu, Lixing Lao, JianPing Chen, and Mingxiao Yang conceived and composed the protocol. Ling Yi, Linna Bo, and Hongzhi Tang majorly participated in enrollment and clinical treatment. Jie Yang and Fanrong Liang designed the trial. Hui Zheng calculated the sample size and developed the data analysis process.

Acknowledgments

This trial is financially supported by research grants from National Key Basic Research Program, China 973 Program, Grant no. 2012CB518501; the Science and Technology Department of Sichuan province, Grant nos. 2011SZ0302 and 2012SZ01270; National Natural Science Foundation of China, Grant nos. 81303060 and 81273854; and the Sichuan Province Department of Education Innovation Team, Grant no. 12TD002.

References

[1] L. French, "Dysmenorrhea," *American Family Physician*, vol. 71, no. 2, pp. 285–292, 2005.

[2] B. Thomas and A. Magos, "Modern management of dysmenorrhoea," *Trends in Urology, Gynaecology & Sexual Health*, vol. 14, no. 5, pp. 25–29, 2009.

[3] M. Y. Dawood, "Dysmenorrhea," *Journal of Reproductive Medicine for the Obstetrician and Gynecologist*, vol. 30, no. 3, pp. 154–167, 1985.

[4] A. S. Coco, "Primary dysmenorrhea," *American Family Physician*, vol. 60, no. 2, pp. 489–496, 1999.

[5] A. R. Davis and C. L. Westhoff, "Primary dysmenorrhea in adolescent girls and treatment with oral contraceptives," *Journal of Pediatric and Adolescent Gynecology*, vol. 14, no. 1, pp. 3–8, 2001.

[6] M. A. Campbell and P. J. McGrath, "Non-pharmacologic strategies used by adolescents for the management of menstrual discomfort," *The Clinical Journal of Pain*, vol. 15, no. 4, pp. 313–320, 1999.

[7] J.-H. Chiu, "How does moxibustion possibly work?" *Evidence-Based Complementary and Alternative Medicine*, vol. 2013, Article ID 198584, 8 pages, 2013.

[8] J.-I. Kim, J.-Y. Choi, H. Lee, M. S. Lee, and E. Ernst, "Moxibustion for hypertension: a systematic review," *BMC Cardiovascular Disorders*, vol. 10, article 33, 2010.

[9] M. Okazaki, S. Aizawa, M. Yamauchi, and K. Oguchi, "Effects of single moxibustion on cutaneous blood vessel and microvascular permeability in mice," *The American Journal of Chinese Medicine*, vol. 18, no. 3-4, pp. 121–130, 1990.

[10] H. Yang, C.-Z. Liu, X. Chen et al., "Systematic review of clinical trials of acupuncture-related therapies for primary dysmenorrhea," *Acta Obstetricia et Gynecologica Scandinavica*, vol. 87, no. 11, pp. 1114–1122, 2008.

[11] National Institutes of Health, "ClinicalTrials. gov," 2009.

[12] X. Zhu, A. Bensoussan, L. Zhu et al., "Primary dysmenorrhoea: a comparative study on Australian and Chinese women," *Complementary Therapies in Medicine*, vol. 17, no. 3, pp. 155–160, 2009.

[13] Y. Bu, S. Chen, and G. Du, "Study on the modern application of acupoints for primary dysmenorrhea," *Journal of Traditional Chinese Medicine*, no. 9, pp. 811–819, 2010.

[14] P. Deadman, K. Baker, and M. Al-Khafaji, *A Manual of Acupuncture: Point Cards*, The Journal of Chinese Medicine Publications, 2nd edition, 1998.

[15] C. Cao, C. Liu, L. Huang, W. Chen, and X. Li, "Clinical observation on abdominal acupuncture and moxibustion treatment of primary dysmenorrhea," *China Medical Herald*, no. 22, pp. 128–129, 2010.

[16] R. Zhang, "History and current state of moxibustion," *Journal of Chinese Integrative Medicine*, vol. 2, no. 6, pp. 466–473, 2004.

[17] B. Flaws, *A Handbook of Menstrual Diseases in Chinese Medicine*, Blue Poppy Press, 1st edition, 1997.

[18] J.-E. Park, M. S. Lee, S. Jung et al., "Moxibustion for treating menopausal hot flashes: a randomized clinical trial," *Menopause*, vol. 16, no. 4, pp. 660–665, 2009.

[19] K. R. Shin, S. A. Kwak, J. B. Lee, and H. R. Yi, "The effectiveness of hand acupuncture and moxibustion in decreasing pain and "coldness" in Korea women who have had hysterectomy: a pilot study," *Applied Nursing Research*, vol. 19, no. 1, pp. 22–30, 2006.

[20] R. Zhao, "Acupuncture-moxibustion treatment for polycystic ovary infertility: a report of 20 cases," *Journal of Acupuncture and Tuina Science*, vol. 10, no. 2, pp. 89–91, 2012.

[21] M. L. Furlow, D. A. Patel, A. Sen, and J. R. Liu, "Physician and patient attitudes towards complementary and alternative medicine in obstetrics and gynecology," *BMC Complementary and Alternative Medicine*, vol. 8, article 35, 2008.

[22] M. K. Merboth and S. Barnason, "Managing pain: the fifth vital sign," *Nursing Clinics of North America*, vol. 35, no. 2, pp. 375–383, 2000.

[23] D. J. Cox and R. G. Meyer, "Behavioral treatment parameters with primary dysmenorrheal," *Journal of Behavioral Medicine*, vol. 1, no. 3, pp. 297–310, 1978.

[24] J. R. Dingfelder, "Primary dysmenorrhea treatment with prostaglandin inhibitors: a review," *American Journal of Obstetrics and Gynecology*, vol. 140, no. 8, pp. 874–879, 1981.

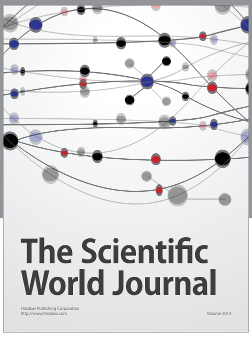

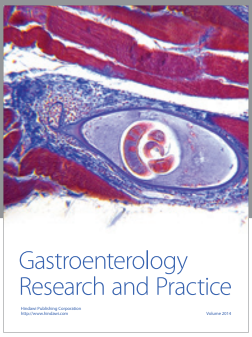

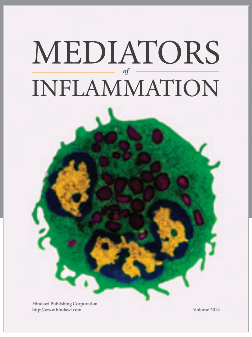

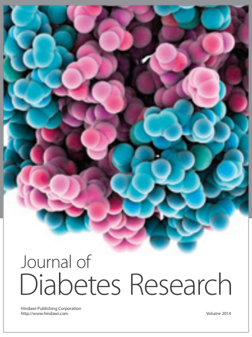

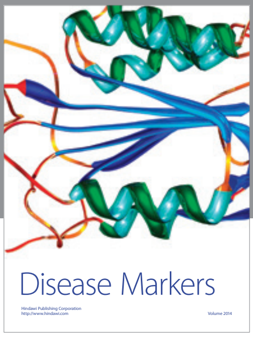

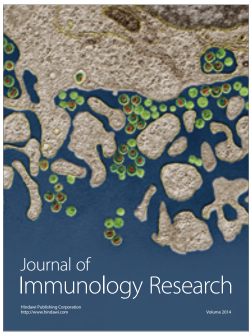

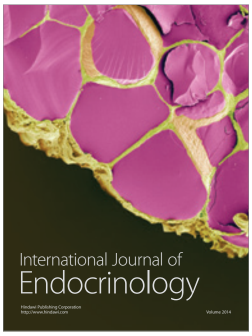

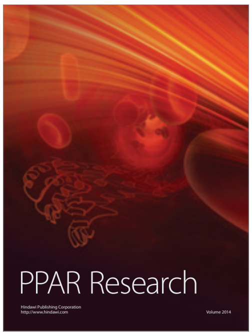

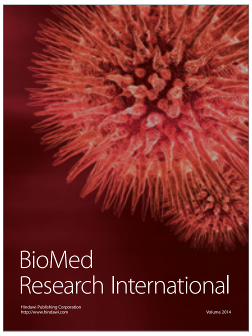

Hindawi

Submit your manuscripts at
http://www.hindawi.com

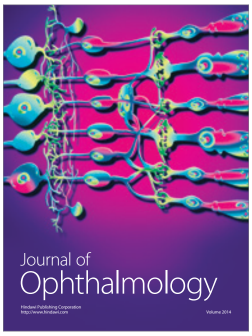

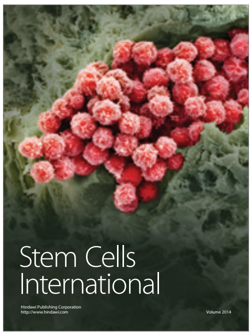

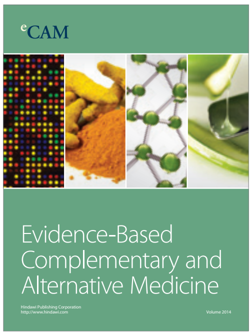

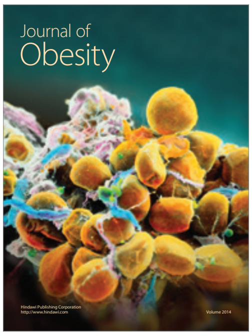

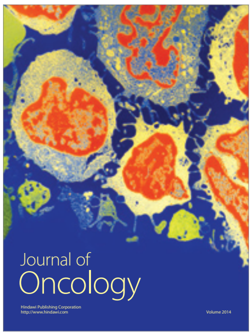

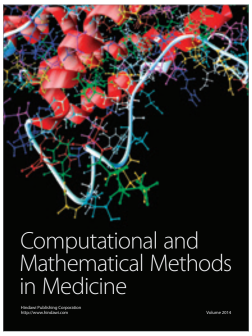

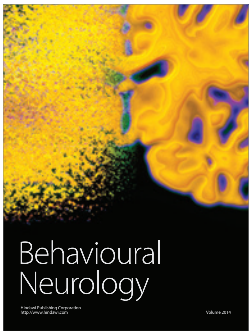

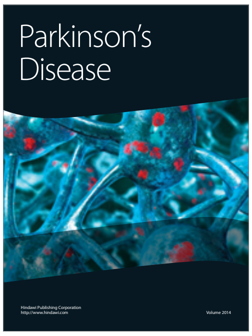

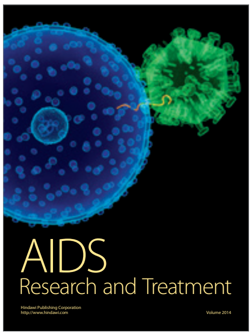

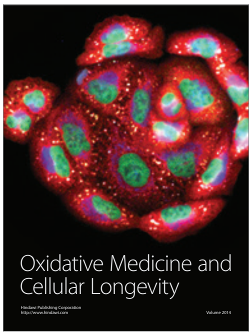

Supplement: S2 File — Published study protocol. (PDF) [file pone.0170952.s005.pdf]
