# Supplementary material for: Moxibustion for pain relief in patients with primary dysmenorrhea: A randomized controlled trial
Source: PLoS One. 2017 Feb 7;12(2):e0170952. doi: 10.1371/journal.pone.0170952 (PMC5295763; doi:10.1371/journal.pone.0170952)
Supplement: S3 File — Results of per-protocol analysis. (DOCX) [file pone.0170952.s006.docx]

**Results of Per-protocol Analysis**

- **Pain intensity measured by the VAS**

| Group | Experimental(n=69) | Control(n=64) | t | Sig(2-tailed) | 95% CI |
| --- | --- | --- | --- | --- | --- |
| Visit0 | 6.34±1.25 | 6.42±1.24 | -0.41 | 0.69 | -0.51, 0.34 |
| Visit1 | 5.41±1.35 | 4.65±1.17 | 3.46 | 0.001 | 0.33, 1.19 |
| Visit2 | 4.21±1.13 | 3.51±1.10 | 3.29 | 0.001 | 0.26, 1.02 |
| Visit3 | 2.26±1.00 | 2.15±0.82 | 0.65 | 0.52 | -0.21, 0.42 |
| Visit4 | 2.43±1.23 | 2.57±1.21 | -0.67 | 0.51 | -0.56, 0.28 |
| Visit5 | 2.55±1.25 | 3.12±1.67 | -2.68 | 0.008 | -0.98, -0.15 |
| Visit6 | 2.84±1.26 | 3.89±1.11 | -5.07 | 0.000 | -1.45, -0.64 |

- **Days with menstrual pain**

| Group | Experimental(n=69) | Control(n=64) | t | Sig(2-tailed) | 95% CI |
| --- | --- | --- | --- | --- | --- |
| Visit0 | 5.47±2.88 | 5.62±2.93 | -0.306 | 0.76 | -1.15, 0.84 |
| Visit1 | 1.68±0.76 | 1.61±0.74 | 0.54 | 0.59 | -0.19, 0.33 |
| Visit2 | 1.37±0.65 | 1.36±1.00 | 0.06 | 0.95 | -0.28, 0.29 |
| Visit3 | 1.12±0.71 | 1.16±1.01 | -0.22 | 0.83 | -0.33, 0.26 |
| Visit4 | 0.85±0.40 | 0.87±0.43 | -0.29 | 0.78 | -0.16, 0.12 |
| Visit5 | 0.92±0.83 | 1.04±0.39 | -2.07 | 0.041 | -0.24, -0.01 |
| Visit6 | 1.06±0.36 | 1.38±0.55 | -4.00 | 0.000 | -0.48, -0.16 |

- **PD symptom severity measured by the CMSS**

| Group | Experimental(n=69) | Control(n=64) | t | Sig(2-tailed) | 95% CI |
| --- | --- | --- | --- | --- | --- |
| Visit0 | 16.23±8.08 | 16.06±6.67 | 0.13 | 0.90 | -2.38, 2.72 |
| Visit1 | 14.23±7.99 | 13.03±5.63 | 0.99 | 0.32 | -1.19, 3.59 |
| Visit2 | 10.74±6.46 | 10.75±5.81 | -0.1 | 0.99 | -2.12, 2.10 |
| Visit3 | 5.72±4.45 | 7.25±4.32 | -1.98 | 0.05 | -3.05, -0.001 |
| Visit4 | 5.47±4.30 | 7.31±4.43 | -2.43 | 0.017 | -3.33,-0.34 |
| Visit5 | 4.61±3.73 | 8.30±4.79 | -4.98 | 0.000 | -5.15, -2.22 |
| Visit6 | 4.20±3.16 | 9.64±4.73 | -7.85 | 0.000 | -6.81, -4.07 |

- **Days with PD symptoms measured by the CMSS**

| Group | Experimental(n=69) | Control(n=64) | t | Sig(2-tailed) | 95% CI |
| --- | --- | --- | --- | --- | --- |
| Visit0 | 21.54±10.96 | 20.59±9.06 | -0.54 | 0.59 | -2.52, 4.41 |
| Visit1 | 16.80±8.93 | 16.06±7.48 | 0.51 | 0.61 | -2.10, 3.57 |
| Visit2 | 12.65±8.68 | 12.78±7.30 | -0.09 | 0.93 | -2.89, 2.63 |
| Visit3 | 6.64±5.79 | 8.47±5.78 | -1.82 | 0.07 | -3.82, 0.16 |
| Visit4 | 6.00±5.18 | 7.88±5.06 | -2.11 | 0.037 | -3.63, -0.12 |
| Visit5 | 4.33±2.98 | 7.70±4.52 | -5.11 | 0.000 | -4.67, -2.06 |
| Visit6 | 3.89±2.67 | 9.17±4.64 | -8.11 | 0.000 | -6.56, -3.99 |

| **Group** | **Experimental(n=69)** | **Control(n=64)** | **t** | **Sig(2-tailed)** | **95% CI** |
| --- | --- | --- | --- | --- | --- |
| **Pain intensity measured by the VAS** | | | | | |
| Visit0 | 6.34±1.25 | 6.42±1.24 | -0.41 | 0.69 | -0.51, 0.34 |
| Visit1 | 5.41±1.35 | 4.65±1.17 | 3.46 | 0.001 | 0.33, 1.19 |
| Visit2 | 4.21±1.13 | 3.51±1.10 | 3.29 | 0.001 | 0.26, 1.02 |
| Visit3 | 2.26±1.00 | 2.15±0.82 | 0.65 | 0.52 | -0.21, 0.42 |
| Visit4 | 2.43±1.23 | 2.57±1.21 | -0.67 | 0.51 | -0.56, 0.28 |
| Visit5 | 2.55±1.25 | 3.12±1.67 | -2.68 | 0.008 | -0.98, -0.15 |
| Visit6 | 2.84±1.26 | 3.89±1.11 | -5.07 | 0.000 | -1.45, -0.64 |
| **Days with menstrual pain** | | | | | |
| Visit0 | 16.23±8.08 | 16.06±6.67 | 0.13 | 0.90 | -2.38, 2.72 |
| Visit1 | 14.23±7.99 | 13.03±5.63 | 0.99 | 0.32 | -1.19, 3.59 |
| Visit2 | 10.74±6.46 | 10.75±5.81 | -0.1 | 0.99 | -2.12, 2.10 |
| Visit3 | 5.72±4.45 | 7.25±4.32 | -1.98 | 0.05 | -3.05, -0.001 |
| Visit4 | 5.47±4.30 | 7.31±4.43 | -2.43 | 0.017 | -3.33,-0.34 |
| Visit5 | 4.61±3.73 | 8.30±4.79 | -4.98 | 0.000 | -5.15, -2.22 |
| Visit6 | 4.20±3.16 | 9.64±4.73 | -7.85 | 0.000 | -6.81, -4.07 |
| **PD symptom severity measured by the CMSS** | | | | | |
| Visit0 | 16.23±8.08 | 16.06±6.67 | 0.13 | 0.90 | -2.38, 2.72 |
| Visit1 | 14.23±7.99 | 13.03±5.63 | 0.99 | 0.32 | -1.19, 3.59 |
| Visit2 | 10.74±6.46 | 10.75±5.81 | -0.1 | 0.99 | -2.12, 2.10 |
| Visit3 | 5.72±4.45 | 7.25±4.32 | -1.98 | 0.05 | -3.05, -0.001 |
| Visit4 | 5.47±4.30 | 7.31±4.43 | -2.43 | 0.017 | -3.33,-0.34 |
| Visit5 | 4.61±3.73 | 8.30±4.79 | -4.98 | 0.000 | -5.15, -2.22 |
| Visit6 | 4.20±3.16 | 9.64±4.73 | -7.85 | 0.000 | -6.81, -4.07 |
| **Days with PD symptoms measured by the CMSS** | | | | | |
| Visit0 | 21.54±10.96 | 20.59±9.06 | -0.54 | 0.59 | -2.52, 4.41 |
| Visit1 | 16.80±8.93 | 16.06±7.48 | 0.51 | 0.61 | -2.10, 3.57 |
| Visit2 | 12.65±8.68 | 12.78±7.30 | -0.09 | 0.93 | -2.89, 2.63 |
| Visit3 | 6.64±5.79 | 8.47±5.78 | -1.82 | 0.07 | -3.82, 0.16 |
| Visit4 | 6.00±5.18 | 7.88±5.06 | -2.11 | 0.037 | -3.63, -0.12 |
| Visit5 | 4.33±2.98 | 7.70±4.52 | -5.11 | 0.000 | -4.67, -2.06 |
| Visit6 | 3.89±2.67 | 9.17±4.64 | -8.11 | 0.000 | -6.56, -3.99 |
